# Dropwort-induced metabolic reprogramming restrains YAP/TAZ/TEAD oncogenic axis in mesothelioma

**DOI:** 10.1186/s13046-019-1352-3

**Published:** 2019-08-09

**Authors:** Claudio Pulito, Etleva Korita, Andrea Sacconi, Mariacristina Valerio, Luca Casadei, Federica Lo Sardo, Federica Mori, Maria Ferraiuolo, Giuseppe Grasso, Anna Maidecchi, Jacopo Lucci, Marius Sudol, Paola Muti, Giovanni Blandino, Sabrina Strano

**Affiliations:** 1 Oncogenomic and Epigenetic Unit, Department of Research, Diagnosis and Innovative Technologies, IRCCS Regina Elena National Cancer Institute, Via Elio Chianesi, 53, 00144 Rome, Italy; 2 Department of Chemistry, University of Rome, “La Sapienza”, Rome, Italy; 3 Policlinico Morgagni, Via del Bosco, 105., Catania, Italy; 4 Aboca Spa, Società Agricola, San Sepolcro, Italy; 5 Department of Physiology, National University of Singapore, Singapore, 117597 Singapore; 6 Department of Oncology, Juravinski Cancer Center, McMaster University, Hamilton, ON L8V5C2 Canada

**Keywords:** HIPPO tumour suppressor pathway, YAP, Mesothelioma, Phytonutrient, Cancer metabolism

## Abstract

**Background:**

Over the past decade, newly designed cancer therapies have not significantly improved the survival of patients diagnosed with Malignant Pleural Mesothelioma (MPM). Among a limited number of genes that are frequently mutated in MPM several of them encode proteins that belong to the HIPPO tumor suppressor pathway.

**Methods:**

The anticancer effects of the top flower standardized extract of *Filipendula vulgaris* (Dropwort) were characterized in “in vitro” and “in vivo” models of MPM. At the molecular level, two “omic” approaches were used to investigate Dropwort anticancer mechanism of action: a metabolomic profiling and a phosphoarray analysis.

**Results:**

We found that Dropwort significantly reduced cell proliferation, viability, migration and in vivo tumor growth of MPM cell lines. Notably, Dropwort affected viability of tumor-initiating MPM cells and synergized with Cisplatin and Pemetrexed in vitro. Metabolomic profiling revealed that Dropwort treatment affected both glycolysis/tricarboxylic acid cycle as for the decreased consumption of glucose, pyruvate, succinate and acetate, and the lipid metabolism. We also document that Dropwort exerted its anticancer effects, at least partially, promoting YAP and TAZ protein ubiquitination.

**Conclusions:**

Our findings reveal that Dropwort is a promising source of natural compound(s) for targeting the HIPPO pathway with chemo-preventive and anticancer implications for MPM management.

**Electronic supplementary material:**

The online version of this article (10.1186/s13046-019-1352-3) contains supplementary material, which is available to authorized users.

## Background

Malignant pleural mesothelioma (MPM) is a rare and aggressive tumor arising from lining of pleural or peritoneal mesothelial cell surfaces [1, 2]. Currently, the clinical outcome is poor with the median survival rate of less than 12 months. Its silent clinical progression and the aberrant resistance to current therapies underlie the poor prognosis of the disease [3, 4]. Platinum based-chemotherapy combined with the folate antagonist, Pemetrexed, represents the conventional treatment [5, 6]. At the present, there is no second line standard therapy for MPM. Consequently, there is an urgent need of new therapeutic options for such a rare if not an orphan disease [7].

There are three histological subtypes of MPM: the epithelioid, the spindled and the biphasic MPM [8]. Most of the MPM incidence at population levels is explained by asbestos exposure after decades of latency period [9–11]. Thus the MPM incidence is expected to rise in the next decade with a large variability across countries due to differences in asbestos commercialization, its use and the duration of banned periods [12–14]. The pathophysiology of the disease is mostly explained by chronic inflammation due to the inhalation of nanoparticles causing mesothelial surfaces infiltration by macrophages that activate pro-inflammatory responses and a release of cytokines and chemokines to pleural and lung tissues [2, 15, 16]. Other factors, including Erionite, a natural fibrous compound that belongs to zeolite minerals, a radiation exposure, an infection by SV40 virus or various genetic factors have been considered as additional risk factors for MPM development [12, 17, 18].

Dietary phytochemicals usually do not cause adverse effects and generally have ability to target multiple signalling pathways. Therefore, medicinal plants have been considered appealing as co-adjuvants in anticancer therapies [19]. Natural polyphenols exhibit pleiotropic anticancer effects thereby impacting on cell proliferation, apoptosis, angiogenesis, oxidation and inflammation [20]. Collectively, it has previously been shown that some phytonutrients can exert anticancer effects in mesothelioma cell lines by interfering with specific oncogenic and/or tumor suppressor pathways [21–25].

The HIPPO signalling pathway controls cell proliferation and organ size [26–28]. YAP and TAZ, are two major effectors of the Hippo pathway and act as sensors of the cell microenvironment and as regulators of cell stemness [29]. Importantly, YAP and TAZ are aberrantly expressed in a wide variety of tumors and their activation drives different steps of the metastatic cascade [30–32]. Despite the fact that the Hippo pathway is deregulated in human cancers, only a few somatic mutations have been reported so far in the Hippo pathway genes. MPM is one of a few cancers that harbor mutations in Hippo pathway genes [33]. Accordingly to the COSMIC database, the genes that are most frequently mutated in MPM are loss of function mutations of tumor suppressor genes such as cyclin dependent kinase inhibitor 2A gene (CDNKN2A), TP53, Neurofibromin 2 (NF2) and BRACA1 associated protein gene 1 (BAP1) [34]. Transcriptome and whole-exome sequencing have revealed that NF2 is frequently mutated in tissues of MPM patients together with a copy number loss and/or loss of expression of NF2, LATS1 and LATS2 [35–40]. NF2 is an upstream regulator of the Hippo pathway. The inactivation of NF2 and LATS2 in MPM prevents the phosphorylation of the transcriptional co-activators YAP/TAZ at Serine 127. This leads to the YAP shuttling into the cell nucleus, favouring an unregulated interaction with TEAD family transcription factors; thereby promoting tumorigenesis [33, 41, 42]. The identification of agents that inhibit YAP/TAZ oncogenic activities might represent a novel and efficacious therapeutic approach for treatment of MPM patients [43–45] Here, we screen thirty natural extracts with anti-inflammatory properties (data not shown) Among them we found that *Filipendula vulgaris* top flower extract exhibited the best IC50 value in MPM cell lines treatment. *Filipendula hexapetala Gilib* (dropwort, syn. *Filipendula vulgaris* Moench) belongs to genus *Filipendula* (*Rosaceae*). It is a perennial herb (up to 80 cm high) diffused in Eastern European countries with pinkish white flowers and characteristic tuberous roots [46]. It is known for its analgesic, antirheumatic, anti-inflammatory and diuretic effects [47, 48]. Several evidences have reported other properties of the plant as antimicrobial, antigenotoxic, antioxidant [49, 50] and hepatoprotective effects [51].

We also show that a standardized extract from Dropwort (*Filipendula vulgaris*, Fil.v.), significantly impairs mesothelioma progression in “in vitro” and “in vivo” models of the disease. This impairment occurs, at least partially, through the silencing of YAP and TAZ oncogenic activities. We find that Dropworth*-*derived formulation promotes YAP/TAZ ubiquitination and therefore restrains the oncogenic axis of YAP/TAZ/TEAD in MPM progression.

## Materials and methods

### Cell cultures and treatments

The human MPM cell lines MPP-89, NCI-2052, MSTO-211H and NCI-H28 were purchased from the ATCC (Rockville, MD). Human mesothelial cells (HMC) were purchased from Tebu-Bio (Le Perray en- Yvelines, France). Mesothelial basal cell medium (Zen-Bio, Research Triangle Park, NC) was used for HMC culture, while MPM cell lines, were cultured in DMEM/F12 + GLUTAMAX (InVitrogen, Carlsbad, CA) supplemented with 10% non-heat inactivated FBS (Gibco, Life Technologies, USA). Fil.v. standardized extract was provided and characterized by Aboca, Sansepolcro, Italy (Table 1) [25]. Dried *Filipendula vulgaris* flower is submitted to hydro-alcoholic extraction with a 50% alcohol ethanol water solution. Extraction is performed at 50 °C for at least 8 h. At the end of the extraction time, flowers are removed from the obtained rich hydroalcoholic solution by filtration. The obtained solution is concentrated by Thin-Film evaporation until the ethanol is removed. Concentrated aqueous solution is dried in a freeze-drier equipment until a solid cake is obtained. Freeze-dried cake is reduced to a powder using a hammer mill and blended to obtain a homogeneous freeze-dried extract powder. A homogeneous sample of each single lot is taken for Quality Control testing. The freeze-dried extract is submitted to a complete characterization of their composition by means of metabolomic analysis (MS-HPLC) and by quantitative analysis of the main chemical classes of compounds (phenols, phenolic acids, flavonoids, lignins, tannins, pheylpropanoid derivatives, salicilates, fat, proteins, amino acids, minerals, polysaccharides) together with the most important single chemical compounds [52]. Freeze-dried extracts were characterized by means of ESI-MS metabolomic fingerprint. In particular, the results of metabolomic analysis by ESI_MS and subsequent statistical evaluation by multivariate analysis for several samples takes into account, emphasized a general maintenance of the characteristics of the product within the period and the condition used. Finally, the extract was prepared by dissolving 50 mg of the plant powder extract in 1 ml of a 50% ethanol solution**.** Pemetrexed (ALIMTA, Eli Lilly and Company, Indiana, USA) and Cisplatin (Pfizer Pharmaceuticals Group, New York, USA) were dissolved according to the manufacturer’s instructions.

**Table 1:** Dropwort (*Filipendula vulgaris*) extract components. Different classes of compounds present in the Dropwort flowering tops phytocomplex are reported. The phytocomplex was derived from *Filipendula vulgaris*

Classes of Compounds		Levels found in Dropwort freeze-dried extract
Polyphenols (%)	total	54.84
	Of which Lignins total	0.466
	Of which tannins total	10.44
	Of which phenols and phenolic acids total	20.36514
	- Gallic acid	9.9563
	- Protocatechuic acid	0.1445
	- Ellagic acid	2.4909
	- Gentistic acid	0.4442
	- 3,4-Dihydroxyphenylglycol	0.39184
	- Methyl gallate (methyl 3,4,5-trhydroxyphenylglycol)	4.519
	- Shikimic acid	2.4184
	Of which flavonoids total	21.7185
	Of wich flavonols, flavanols, isoflavones	21.1935
	- Quercetin	14.35
	- Quercitrin	1.88
	- Quercetin 3-b-glucoside	0.793
	- Quercetin 3-D-galactoside	3.61
	- Kaempferol	0.5536
	Of which catechins	0.525
	Of which phenylpropanoid derivates	1.3069
	- Chlorogenic acids	1.121
	- Caffeic acids	0.1859
	- Of which salicilates total	0.5435
Organic Acids (%)	total	5.757
Protein (%)	total	6.1079
Free amino acids (%)	total	4.64
Polysaccharides (%)	total	5.1
	Of which Soluble dietary fiber	2.6
	Of which Insoluble dietary fiber	2.5
Saccharides (%)	total	3.41
	Of which monosaccharides	
	Fructose	1.49
	Glucose	1.92
Fats (%)	total	0.507
Minerals (*Oligo, micro and macroelements*) (%)	total	3.598

### RNA processing and qRT-PCR

Total RNA from mesothelioma cell lines differently treated or not was extracted by using Trizol Reagent following manufacturer’s instructions (Ambion). cDNA was synthesized according to the manufacturer’s instructions (M-MLV RT kit, Invitrogen). Gene expression was measured by real-time PCR using the FastStart SYBR Green Master Mix (Applied Biosytems) on a studio 7-instrument (Applied Biosystems). Sequences of qPCR primers are ACTIN Fw: 5′-GGCATGGGTCAGAAGGATT-3′, Rv: 5′-CACACGCAGCTCATTGTAGAAG-3; YAP1 Fw: 5′-CACAGCATGTTCGAGCTCAT-3′, Rv: 5′-GATGCTGAGCTGTGGGTGTA-3′; TAZ Fw: 5′-CCATCACTAATAATAGCTCAGATC-3′, Rv: 5′-GTGATTACAGCCAGGTTAGAAAG-3′; MCM7 Fw: 5′-TCGAGGCATGAAAATCCGGG-3′, Rv: 5’CGCCAGTCGATCAATGTATGACA-3′; ANKRD1 Fw: 5′-AGTAGAGGAACTGGTCACTGG-3′, Rv: 5′-TGGGCTAGAAGTGTCTTCAGAT-3′; CTGF Fw: 5′-GCCACAAGCTGTCCAGTCTAATCG-3′, Rv: 5′-TGCATTCTCCAGCCATCAAGAGAC-3′; p21 Fw: 5′-GGGACAGCAGAGGAAGAC-3′, p21 Rv: 5′-GCGTTTGGAGTGGTAGAAATC-3′.

### Cell viability assay

Cell viability of treated cells was assessed using ATPlite assay (Perkin Elmer, Massachusset, USA) accordingly to the manufacturer’s instructions. Cells (8 × 10^2^ cells) were seeded in 96 well-plates and cultured for 24 h and treated for 72 h with Fil.v. extract (0, 3, 6, 12.5, 25, 50, 100 and 200 μg/ml). Each plate was evaluated immediately on a microplate reader (Expire Technology, Perkin Elmer). Calcusyn software was used to calculate combination index (CI) [53].

### Clonogenic assays

MPM cell lines were grown at 70% confluence and treated with Fil.v. extract or with vehicle. Sixteen hours later, cells were detached and seeded at 600 cells per 6 well into six-well dishes (Corning-Costar, Tewksbury, MA, USA) in drug-free media. Fresh media (25%) was added every three days. After 15–21 days, colonies were stained with crystal violet and colonies counted.

### Apoptosis detection

For propidium iodide (PI) staining, cells were seeded in 6-well plates at a density of 10^4^ cells/ml. After 24 h cells were treated with indicated plant extract concentrations for different time intervals. Floating and attached cells were harvested, washed in PBS, fixed in ice-cold ethanol (70% v/v) and stored at − 20 °C. For the analysis, cells were washed in PBS and incubated with RNase A (1 mg/ml) and PI (40 μg/ml) was added. For PI/Annexin V double staining treated cells were harvested and suspended in binding buffer (HEPES pH 7.4, CaCl_2_ 2.5 mM, NaCl 140 mM). Aliquots of cells were incubated for 15 min with Annexin V FITC (0.2 μg/ml) (Abcam, ab-63,556) and PI (5 mg/ml) (Invitrogen). For each FACS analysis, 3 × 10^3^ events for each sample were analyzed. Flow cytometry analyses were carried out with Easycyte 8HT (Guava, Millipore) followed by analysis using InCyte software (Millipore).

### ALDH activity assay

ALDEFLUOR kit (Stem Cell Technologies, Vancouver, Canada) was used to asses ALDH activity of MSTO-211H treated or not with Fil.v. extract. ALDH-positive cells showed greater fluorescence compared to a control staining reaction containing the ALDH inhibitor, DEAB (diethylaminobenzaldehyde), upon addition of the synthetic ALDH substrate BAAA. Flow cytometry analyses were carried out with Easycyte 8HT (Guava, Millipore) followed by analysis using InCyte software (Millipore).

### Transwell invasion assay

Migration assay was performed using a 24-well Boyden chamber with a non-coated 8-mm pore size filter in the insert chamber (BD Falcon, Franklin Lakes, NJ, USA). Cells were suspended in 0.5 ml DMEM/F12 media without containing FBS and seeded into the insert chamber. Cells were allowed to migrate for 24 h into the bottom chamber containing 0.5 ml of DMEM/F12 media containing 10% FBS in a humidified incubator at 37 °C in 5% CO_2_. Migrated cells that attached to the outside of the filter were visualized by staining with DAPI (Thermo Fisher) and counted. The average number of cells per field was expressed as percentage of the control after normalizing for cell number.

### Wound–healing migration assay

MPM cells were grown to 80% of confluence in 6-well tissue culture plates and wounded with a sterile 10-ml pipet tip to remove cells. PBS washing was used to remove loosely attached cells. The progression of migration was photographed at different under a light microscope. The number of cells migrated into the scratched area was calculated.

### Western blot analysis and protein immunoprecipitation

Cell lysis was performed on ice for 30 min in NP40 lysis buffer (50 mM Tris-HCl pH 7.4, 150 mM NaCl, 1% NP-40, 1 mM EGTA, 1 mM EDTA) supplemented with protease and phosphatase inhibitors (5 mM PMSF, 3 mM NaF, 1 mM DTT, 1 mM NaVO_4_). Equal amounts of total proteins extracts (10–30 μg) were resolved by 8–12% denaturing SDS polyacrylamide gel electrophoresis (SDS-PAGE), and transferred for 1 h and 30 min to polyvinylidene difluoride membrane. Membranes were blocked in 5% milk-TBS-0.05% Tween 20 for 1 h and incubated overnight with the specific primary antibodies. In the co-immunoprecipitation the lysis buffer was modified accordingly the protein isoelectric point. Protein concentrations were determined by colorimetric assay (Bio-Rad, Hercules, CA, USA). For each immunoprecipitation, 1 μg of rabbit YAP (Santa Cruz, sc-15,407) or mouse TAZ antibody (Sigma Aldrich, T4077) and 1 μg of rabbit or mouse IgG (Santa Cruz Biotech, sc66931 and sc69786) as control were used. Pre-cleared extracts were incubated with protein A/G-Agarose beads (Thermo Fisher Scientific, Rockford, IL, USA) in lysis buffer containing 0.05% BSA and antibodies, under constant shaking at 4 °C for 3 h. After incubation, agarose bead-bound immunocomplexes were rinsed with lysis buffer and eluted in 50 ml of SDS sample buffer for western blotting.

The following primary antibodies were used: anti phospho-AMPKα (Thr-172) (Cell Signaling, #2531); anti-phospho-mTOR (Ser-2448) (Cell Signaling, #2971); anti- β Actin (Santa Cruz, sc-81,178); anti-phospho p70 S6 Kinase (thr-389) (Cell Signaling, #9234); anti-phospho S6 (ser235/236) (Cell Signaling, #2211); PARP (Cell Signaling, # 9542); anti-caspase 7 (Cell Signaling, #9492); anti-caspase 3 (Enzo life Science, #31A1067); anti YAP (Santa Cruz, sc-15,407); anti TAZ (Sigma Aldrich, T4077); anti GAPDH (Santa Cruz, sc-47,724); anti-TEAD1 (BD, cat.n.-610,923); anti H1 (Cell Signaling, #41328) anti Tubulin (Abcam, Ab44928); anti MCM7 (Cell Signaling, #3735); anti HA (Santa Cruz, sc-57,592). All the indicated antibodies were used at the minimum dilutions suggested by the manufacturer. Secondary horseradish peroxidase-conjugated was purchased from Santa Cruz. ECL reagent (Amersham, GE Healthcare, Piscataway, NJ, USA) was employed for the chemo-luminescence detection. The Uvitec Alliance software (Eppendorf) was used to quantify the obtained data.

### Nucleo/cytosol extracts preparation

MSTO-211H treated cells were washed in cold PBS. After pelleting cells were lysed in 10 mM HEPES-KOH pH 7.9, 1.5 mM MgCl_2_, 10 mM KCl, 0.5 mM dithiothreitol, 0.2 mM phenylmethylsulfonyl fluoride for 10 min. After centrifugation, supernatants were removed and represented cytosolic fraction, while the pellet (nuclear fraction) was treated in a high salt buffer (20 mM HEPES-KOH pH 7.9; 25% glycerol; 0.42 M NaCl; 1.5 mM MgCl_2_; 0.2 mM EDTA; 0.5 mM dithiothreitol; 0.2 mM phenylmethylsulfonyl fluoride) for 20 min to extract nuclear proteins [54].

### Ubiquitination assay

A number of 1.6 × 10^6^ cells was transfected with a pCMV vector carrying hemagglutinin (HA)-tagged ubiquitin (Ub-HA) [26]. After 18 h, cells were treated with 25 μM MG-132 for a further 6 h. Protein extracts were immunoprecipitated as described and subjected to Western Blotting.

### Plasmids and transfections

Transfections were performed with Lipofectamine 2000 or Lipofectamine RNAiMax (Life Technologies) according to manufacturer’s recommendations. The following siRNAs (Eurofins MWG) were used to inhibit YAP, TAZ and TEAD expression in MSTO-211H cells: siYAP: 5′-GACAUCUUCUGGUCAGAGA-3′, siGFP: 5′-AAGUUCAGCGUGUCCGGGGAG-3′, siTAZ is a pool of two independent siRNAs mixed in equal amount: 5′-AAAGUUCCUAAGUCAACGU-3′ and 5′- AGGUACUUCCUCAAUCACA-3′, si-TEAD: 5′- CGAUUUGUAUACCGAAUAA. The following vectors were used to over express the same genes in MSTO-211H cells: pCDNA3-YAP-Flag, pCS2-TAZ-Flag, pQCXIH-myc-TEAD kindly gifted by prof. Georg Halder.

### Immunofluorescence microscopy

MSTO-211H cells were seeded into eight-chamber culture slides (BD Falcon). The next day, cells were rinsed with ice-cold PBS buffer and fixed with 4% paraformaldehyde for 10′ at room temperature and then permeabilized with 1% Triton X-100. Cells were incubated overnight with the indicated antibody. The day after, cells were washed with cold PBS three times for 3 min each and stained for 2 h with a secondary antibody Alexa 488-conjugated goat anti-mouse IgG or anti-rabbit IgG (Molecular Probes Cells) and counterstained with DAPI (40,6-diamidino-2-phenylindole dihydrochloride). Cells were examined under a Zeiss LSM 510 laser scanning fluorescence confocal microscope (Zeiss, Wetzlar, Germany).

### Sample preparation for NMR spectroscopy

Each medium sample (2 ml) was lyophilized then dissolved in 700 μl of 1 mM TSP [sodium salt of 3-(trimethylsilyl) propionic-2,2,3,3-d4 acid], 10 mM sodium azide D_2_O phosphate buffer solution (pH = 7.4) and finally homogenized by vortex mixing for 1 min. After centrifugation (10 min, 10.000 RCF at 22 °C), 600 μl of each resulting supernatant was transferred to a 5-mm NMR tube and used for the NMR analysis. Cell pellets were lyophilized, weighted and cryogenically grounded using Cryomill (Retsch GmbH Germany) before methanol/chloroform/water extraction (2/2/1.8) to extract polar and non-polar metabolites. Polar extracts were dissolved in 600 μl of 1 mM TSP ((trimethylsilyl)-propionic-2,2,3,3-d4 acid) and 10 mM NaN_3_ solution in D_2_O 0.1 M phosphate buffer (pH = 7.4), while nonpolar extracts were dissolved in 600 μl of CDCl_3_ containing 0.03% TMS (tetramethylsilane)/CD_3_OD solution (2:1, v/v).

### ^1^H-NMR spectroscopy

All 2D 1H J-resolved (JRES) NMR spectra were acquired on a 500 MHz VNMRS Varian/Agilent spectrometer (Agilent, Santa Clara, CA) at 25 °C using a double spin echo sequence with pre-saturation for water suppression and 16 transients per increment for a total of 32 increments. These were collected into 16 k data points using spectral widths of 8 kHz in F2 and 64 Hz in F1. Each free induction decay (FID) was Fourier transformed after a multiplication with sine-bell window functions in both dimensions. JRES spectra were tilted by 45°, symmetrized about F1, referenced to lactic acid at δH = 1.33 ppm and the proton-decoupled skyline projections (p-JRES) exported using Agilent VNMRJ 3.2 software. The exported p-JRES were aligned, corrected for baseline offset and then reduced into spectral bins with widths ranging from 0.02 to 0.06 ppm by using the ACD intelligent bucketing method (1D NMR Manager software, ACD/Labs, Toronto, Canada). This method sets the bucket divisions at local minima (within the spectra) to ensure that each resonance is in the same bin throughout all spectra. The area within each spectral bin was integrated and, to compare the spectra, the integrals derived from the bucketing procedure were normalized to the total integral region. Metabolites were identified using an in-house NMR database and literature data and confirmed by 2D homo- and hetero-nuclear NMR spectroscopy.

### NMR spectra pre-processing treatment

The 1D skyline projections exported were aligned and then reduced into spectral bins with ranging from 0.01 to 0.02 ppm by using the ACD intelligent bucketing method (1D NMR Manager software (ACD/Labs, Toronto, Canada). To compare the spectra, the integrals derived from the binning procedure were normalized to the total integral region, following exclusion of bins representing the residual water peak (4.33–5.17 ppm) and the TSP peak (0.5–0.5 ppm). The resulting data was used as input for multivariate analysis: Principal Component Analysis (PCA and Orthogonal projections to latent structures discriminant analysis (OPLS-DA) were performed using SIMCA-P + version 12 (Umetrics, Umea, Sweden).

### Phospho-protein profiling

The Phospho Explorer antibody microarray, which was designed and manufactured by Full Moon Biosystems, Inc. (Sunnyvale, CA), contains 1318 antibodies. Each of the antibodies has two replicates that are printed on a coated glass microscope slide, along with multiple positive and negative controls. The antibody array experiment was performed using Full Moon Biosystems, according to their established protocol. In brief, cell lysates obtained from MSTO-211H treated with the Fil.v. (50 μg/ml) or vehicle for 24 h, were biotinylated with the antibody array assay kit (Full Moon Biosystems, Inc.). The antibody microarray slides were first blocked with a blocking solution (Full Moon Biosystems, Inc.) for 30 min at room temperature, rinsed with Milli-Q grade water for 3–5 min. The slides were then incubated with the biotin-labeled cell lysates in coupling solution (Full Moon Biosystems, Inc.) at room temperature for 2 h. The array slides were washed 4 to 5 times with 1x Wash Solution (Full Moon Biosystems, Inc.) and rinsed extensively with Milli-Q grade water before detection of bound biotinylated proteins using Cy3-conjugated streptavidin. Each slide (containing six replicates) was hybridized and Cy3 fluorescence acquired by microarray scanner with a scan resolution of 10 mm (Agilent Technologies). The images were quantified using Agilent Feature Extraction (AFE) software (Agilent Technologies). The fluorescence signal of each antibody was obtained from the fluorescence intensity of this antibody spot after subtraction of the blank signal (spot in the absence of antibody) [55].

### Statistical analysis

Bioinformatic analysis was performed with Matlab (The MathWorks Inc.). Z score transformation was used to express the background corrected spot intensity values as unit of a standard deviation from the normalized mean of zero. Features were selected basing on Z ratios calculated by taking the difference between the averages of the observed protein Z scores and dividing by the standard deviation of all the differences for that particular comparison. A Z-ratio that was higher than 1.96 was inferred as significant. Unsupervised Hierarchical Clustering was used to investigate clusters of samples. Pathway analysis was performed by DAVID program.

### EnSpire® cellular label-free platform

MSTO-211H cells were seeded in specially designed 384- well plate with highly precise optical sensors able to measure changes in light refraction resulting from dynamic mass redistribution (DMR) within the cell’s monolayer. Change in the light refraction was indicated by a shift in wavelength.

### Animal studies

CD1 mice were subcutaneously transplanted with MSTO-211H (2 × 10^6^). At the evidence of tumor appearance (when tumor volume reached 60 mm^3^) animals were randomly divided into five groups. Drinking water and a complete pellet diet (GLP 4RF21, Mucedola) were supplied ad libitum. Dueto the complexity of the Fil.v. extract it was not possible to titer the effective compound but rather refer to the total extract amount administrated. Besides, treatment concentrations were translated from in vitro studies. Accordingly, three groups of mice (*n* = 6) were given Fil.v. extract in drinking water at the following concentrations: 25, 50 and 75 mg/ml, whereas mice of the control group (*n* = 6) were given vehicle (daily for three weeks). An average of 8 ml/day was estimated to be drunk by each mouse. In the last group, Pemetrexed was injected intraperitoneally at the dose of 100 mg/kg for five consecutive days [56]. Body weight and clinical signs of the mice were checked every 3 days. After 24 days mice were euthanized while under deep anesthesia and unresponsive to all stimuli. Animals were free of pathogens according to FELASA recommendations and health status was monitored daily. All tumorigenicity assays were performed according to the guidelines set by the internal ethical committee. At the end of the experiment tumor masses were collected and fixed in 10% buffered formalin.

### Xenograft transplantation

MSTO-211H cells were pre-treated with Fil.v. extract (50 μg/ml) for 24 h. Suspensions of 2 × 10^6^ MSTO-211H cells x mouse (*n* = 6) were subcutaneously injected in PBS 1x/Matrigel (BD Biosciences San Jose, CA, USA) into 6-weeks-old female CD1 mice (Charles River, Milan). Tumor volume was calculated by using the formula: V 1/2 x length x width^2^ (by electronic caliper).

### Immunoistochemical analysis

Formalin-fixed and paraffin-embedded 5 μm sections from mice tumor sections were stained with haematoxylin and eosin or stained with anti-ki67 antibody (ab15580, Abcam). Seven fields chosen randomly from each sample were scored.

## Results

### A standardized extract from dropwort inhibits cell proliferation and impairs migration and invasion of MPM cells

**Fig. 1:**
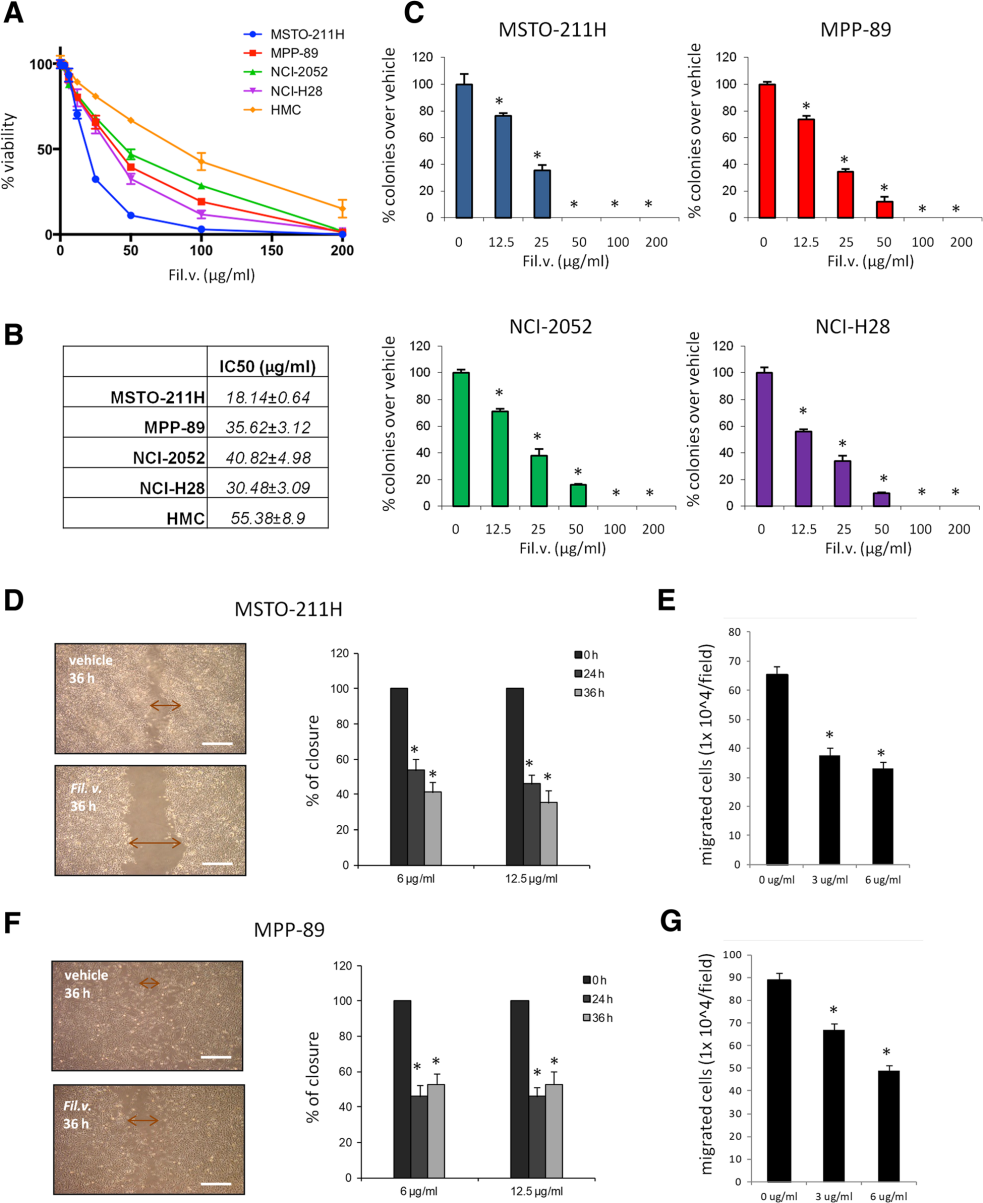
Dropwort extract affects viability and impairs migration and invasion of MPM cells. **a** Viability of MPM cell lines (MSTO-211H, MPP89, NCI-H28 and NCI-2052) and normal untransformed mesothelial cells (HMC) treated for 72 h with different doses of Fil.v. extract (0-200 μg/ml). **b** IC-50 value calculated by Compusyn software. Data are represented as mean +/− SD. Statistics (t-test): *p* < 0.05. **c** Colony forming assay. MSTO-211H, MPP89, NCI-H28 and NCI-2052 cells were treated with different doses of Fil.v. extract (0-200 μg/ml). Histograms showing average colony counts from triplicate experiments. Bars indicate the average of three independent experiments. Statistics (t-test): *p* < 0.05. **d**, **f** Left side: representative micrographs of wound healing closure assays from MSTO-211H (**d**) and MPP-89 (**f**) cells treated for 36 h with 12.5 μg/ml of Fil.v. extract. Right side: histogram showing the healing closure efficiency of the cells treated with vehicle or the Fil.v. extract (6 μg/ml and 12 μg/ml) after different times of treatment (0, 24 and 36 h). Bars indicate the average of three independent experiments. Statistics (*t*-test): *p* < 0.05. **e**, **g** Percentage of invading cells over vehicle. MSTO-211H cells (**e**) or MPP-89 cells (**g**) were treated at the indicated doses of extract. Error bars represent mean +/− SD. Statistics (*t*-test): *p* < 0.05

To find a new treatment for malignant pleural mesothelioma, we tested the effect of the standardized extract from Dropwort plant (referred from now on to as “Fil. v. extract”), on MPM cells viability. To this end, we treated different MPM cell lines (MSTO-211H, MPP-89, NCI-H28, NCI-2052 and untransformed mesothelial cells, HMC) for 72 h with different doses of Fil. v. extract (3, 6, 12, 25, 50, 100 and 200 μg/ml) (Fig. 1a). Subsequently, we measured the half-maximal concentration of growth inhibition (IC50) (Fig. 1b). We found that the Fil.v. extract affected MPM cells viability in a dose dependent manner. HMC cells were more resistant to the Fil. v. extract-induced growth inhibition compared to the MPM cells (Fig. 1a-b); thereby suggesting that the extract is less toxic for mesothelial cells than for their malignant counterparts. Moreover, the Fil.v. extract affected viability of different cancer cell lines that represented different cancer types (Additional file 1: Figure S1c). Next, we tested whether the Fil.v. extract could impair the ability of MPM cells to form colonies. We found that the Fil.v. extract inhibited the capability of MSTO-211H MPP-89, NCI-2052 and NCI-H28 cells to form colonies in a dose dependent manner (Fig. 1c). The induction of apoptosis represents a leading pathway through which many anti cancer therapeutic agents exert their effects. Therefore, we analyzed the cell cycle (Additional file 1: Figure S1a-b) by cytofluorimetry and the presence of phosphatidylserine (PS) on the cell surface by Annexin V labeling (Additional file 1: Figure S1d-e) in MSTO-211H and MPP-89 cells treated or not with different doses of Fil.v. extract. We found that it induced an increase of subG1 peak (Additional file 1: Figure S1a-b) and an increase in the number of Annexin V-positive cells (Additional file 1: Figure S1d-e). Both changes were dose dependent in both cell lines tested. Furthermore, Fil.v. extract at a concentration of 100 μg/ml induced an increase in the cleaved protein levels of different apoptotic markers such as Caspase 3, Caspase 7 and PARP (Additional file 1: Figure S1f) in MSTO-211H cells. Next, we investigated whether sub-apoptotic doses of Fil.v. extract could impair migration and invasiveness of MPM cell lines. To this end, we first performed a scratch wound closure assay in MSTO-211H (Fig. 1d) and MPP-89 (Fig. 1f) cells treated with 6 or 12 μg/ml of the extract. We found that the plant extract inhibited migration of MSTO-211H and MPP-89 cells in a time dependent manner (Fig. 1d, f). We then assessed the invasion capability of MSTO-211H (Fig. 1e) and MPP-89 (Fig. 1g) cells using a 24-well chamber with a non-coated 8-mm pore size filter in the presence of 6 or 12 μg/ml of Fil.v. extract. We found that the extract inhibited in a dose dependent manner the invasion of treated cells compared to those untreated (Fig. 1e, g).

### Dropwort impairs MPM ALDH-bright positive cells viability and affects in vivo tumor growth

**Fig. 2:**
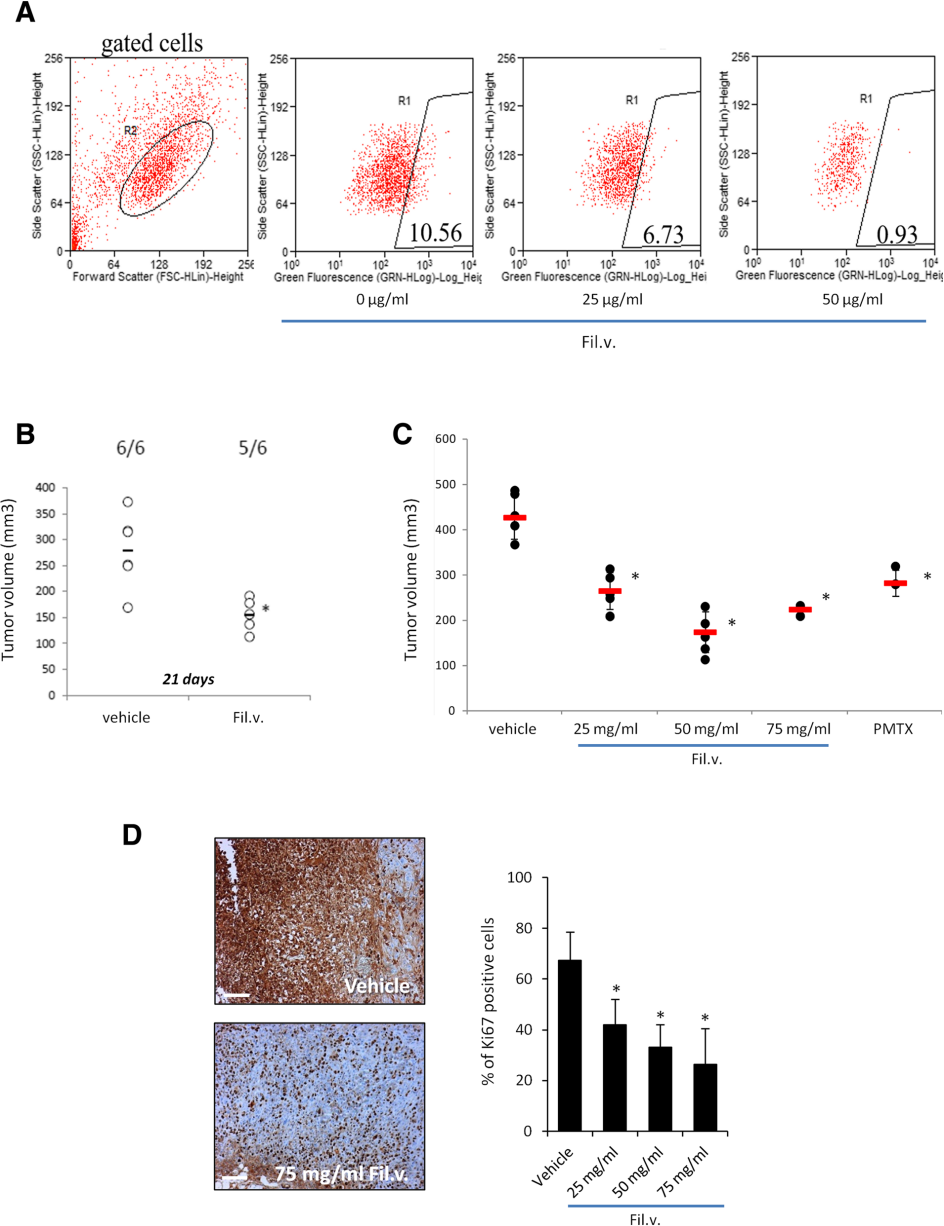
Dropwort extract affects in vivo mesothelioma tumor growth and impairs the survival of chemo resistant subpopulation (ALDH ^bright^ cells) of MPM cells. **a** Fil.v. extract reduces the number of ALDHbright cells in MSTO-211H culture. Representative flow cytometry plots showing the percentage of ALDHbright cells (gated) in MSTO-211H cell cultures treated for 24 h with vehicle or Fil.v. extract (25 μg/ml and 50 μg/ml) and stained for ALDH activity. The percentage of ALDHbright cells was determined over the same cells treated with a specific ALDH inhibitor (DEAB) immediately after adding the ALDH substrate (BAA) (**b**) Suspensions of 2 × 10^6^ MSTO-211H cells were pre-treated with either vehicle or Fil.v. extract (50 μg/ml) for 24 h and subcutaneously injected into CD1 mice. Horizontal bars represent the average tumor volume of the vehicle (*n* = 6) and the Fil.v. extract (*n* = 6) treated engrafted tumors. Tumors were collected 24 days after MSTO-211H cells injection. Statistics (t-test): *p* < 0.05. **c** Fil.v. extract beverage inhibits in vivo mesothelioma tumor progression**.** Tumour volumes of mice (*n* = 6) treated with vehicle, or Pemetrexed or the Fil.v. extract in drinking water are reported. Statistics (t-test): *p* < 0.05. (**d**) Left side. Representative micrographs of the excised tumours stained with anti-Ki-67 antibody. Scale bar, 100 μm. Right side. Histograms show the percentage of Ki-67 positive nuclei scored for each tumor Statistics (t-test): *p* < 0.05

Chemoresistance is the leading cause of poor prognosis for MPM patients. This phenomenon is also due to the presence of cancer stem-like sub-population of cells which undergo Epithelial-To-Mesenchymal Transition (EMT) and exhibit high levels of Aldehyde Dehydrogenase (ALDH) activity [3]. Accordingly, we tested whether the Fil.v. extract could affect ALDH-bright positive cells viability. To this end, MSTO-211H cells were treated with either 0, or 25 or 50 μg/ml of Fil.v. extract for 24 h and stained with ALDEFLUOR kit. ALDH activity levels were measured by using flow cytometer. MSTO-211H untreated cells showed a 10% of ALDH-bright positive cells (Fig. 2a). We noted that the Fil.v. extract treatment affected the viability of this subpopulation of cells also in a dose dependent manner (Fig. 2a). Based on the previous “*in vitro* data”, we tested the Fil.v. extract antitumoral activity also “in vivo”. At first we checked whether the extract treatment could impair the engraftment of MSTO-211H cells injected into CD1 mice. Accordingly, MSTO-211H cells were treated either with vehicle or 50 μg/ml Fil.v. extract for 24 h. Next, pre-treated cell suspensions were injected into CD1 mice and their growth was measured. As suspected, Fil.v. extract-treated cells engrafted less efficiently when compared to controls (Fig. 2b). Further, we evaluated the ability of the natural extract to inhibit growth of xenografted mesothelioma MSTO-211H cells subcutaneously transplanted into CD1 mice. After three weeks of treatment with the Fil.v. extract the tumor xenograft growth was inhibited in a dose dependent manner (Fig. 2c). Interestingly, the treatment of mice with Pemetrexed resulted in a tumor growth reduction similar to those treated with the Fil.v. extract (Fig. 2c). In addition, we analyzed the proliferation rate of the different xenografted tumors by checking their Ki67 gene expression levels. All tumors xenografted into mice that belonged to Fil.v. extract-treated groups exhibited a reduction of more than 30% in the Ki67 expression levels compared to the untreated mice (Fig. 2d).

### Dropwort extract sensitizes MSTO-211H cell to cisplatin or Pemetrexed-induced cell killing

**Fig. 3:**
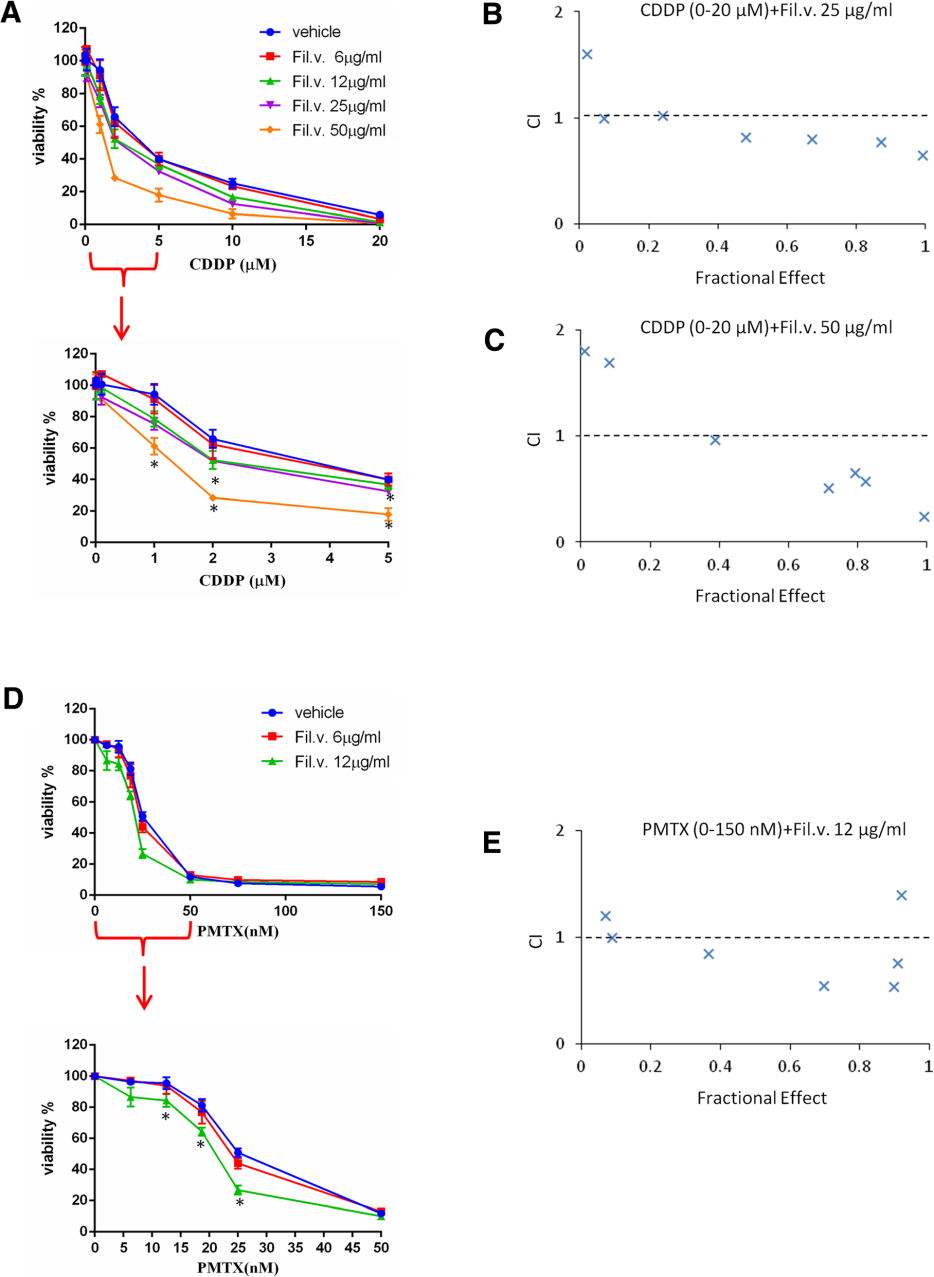
Dropwort treatment sensitizes MSTO-211H cells to MPM conventional chemotherapy. **a** Upper panel. Viability of MSTO-211H cells pre-treated for 24 h with different doses of Fil.v. extract (6–50 μg/ml) and then treated for 72 h with different doses of CDDP (0–20 μM). Lower panel. Zoom on 0–5 μM CDDP treatment range. Data are represented as mean +/− SD. Statistics (t-test): *p* < 0.05. **b** and **c** Combination index (CI) versus the fractional effect obtained from MSTO-211H cells pre-treated with either 25 μg/ml (**b**) or 50 μg/ml (**c**) of Fil.v. extract followed by CDDP treatment (0–20 μM) for 72 h. **d** Upper panel. Viability of MSTO-211H cells pre-treated for 24 h with different doses of Fil.v. extract (6–12 μg/ml) and then treated for 72 h with different doses of PMTX (0–150 nM). Lower panel. Zoom on 0–50 nM PMTX treatment range. Data are represented as mean +/− SD. Statistics (t-test): *p* < 0.05. **e** Combination index (CI) versus the fractional effect obtained from MSTO-211H cells pre-treated with 12 μg/ml of Fil.v. extract followed by PMTX treatment (0–150 nM) for 72 h

As reported before, platinum based-chemotherapy and Pemetrexed represent the standard therapy for mesothelioma management [5, 6]. Unfortunately, MPM exhibits resistance to this chemotherapy after few months of treatment [57, 58]. Treatment of MPM with Cisplatin (CDDP) plus Pemetrexed (PMTX), in fact, increases patient survival by only a few months [59, 60]. Consequently, we tested whether Filipendula extract could sensitize MPM cells to CDDP or PMTX treatment. To this end we have pre-treated MSTO-211H cells with either vehicle or different doses of Fil.v. extract (6, 12, 25 and 50 μg/ml) for 24 h and subsequently treated with CDDP (0–20 μM) and PMTX (0–150 nM) for 72 h (Fig. 3). We found that Fil.v. extract sensitized MSTO-211H mesothelioma cell lines to CDDP (Fig. 3a-c) and Pemetrexed-induced cell killing (Fig. 3d, e).

### Dropwort affects multiple pathways in mesothelioma cells

**Fig. 4:**
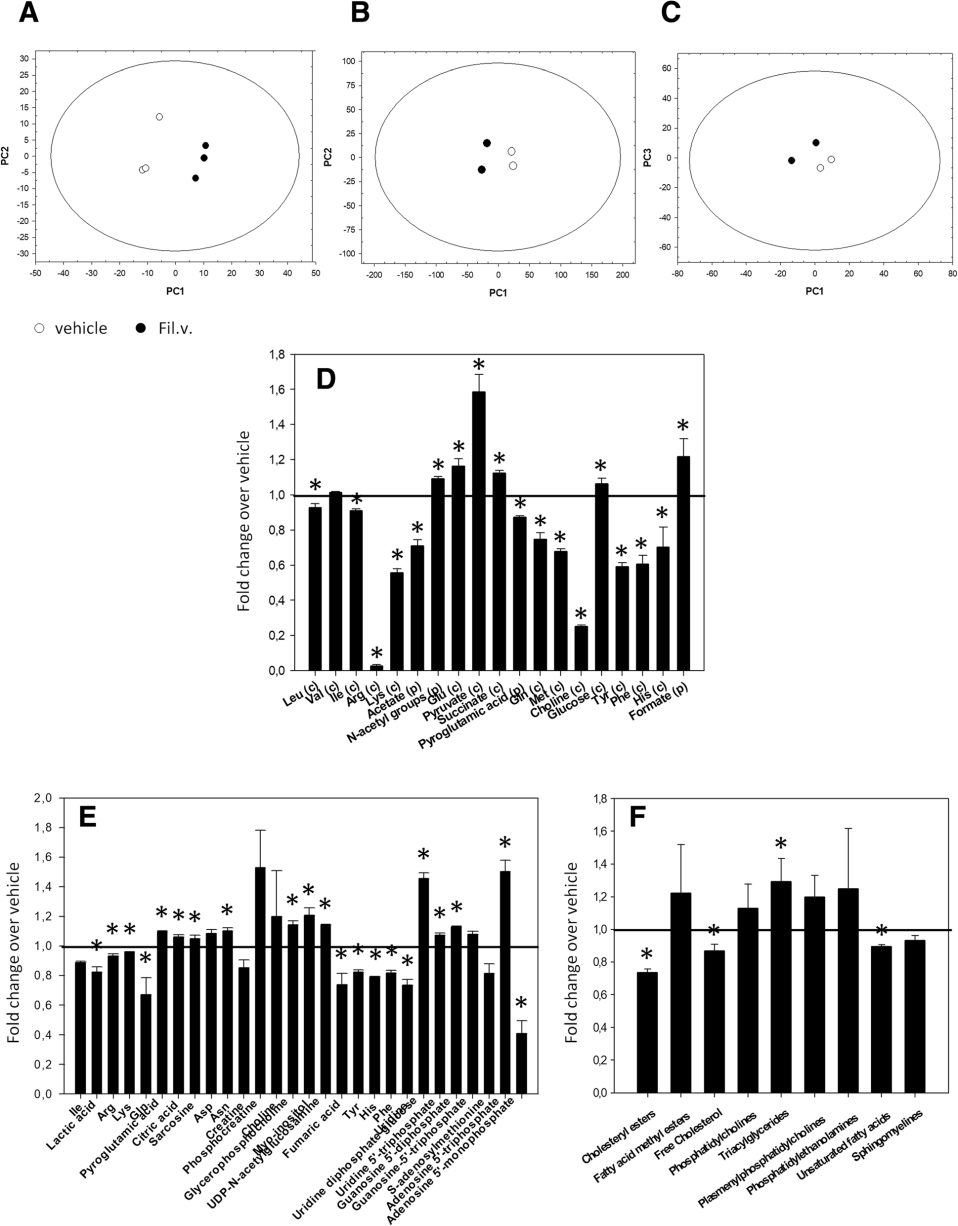
Dropwort treatment induces a perturbation in the metabolic profile of MSTO-211H cell lines. **a**-**f** Metabolic response of MSTO-211H cell lines to Fil.v. extract treatment. PCA models built on the ^1^H-NMR dataset of media samples (**a**) and hydrophilic (**b**) and lipophilic (**c**) cell extracts from Fil.v. extract-treated and vehicle-treated MSTO-211H cell cultures. The score plots show metabolic differences between the two cell groups (*p* = 0.001 and *p* = 0.01 on PC1 for models in A e B respectively and linear discriminant analysis on PC1 and PC3 for model in (**c**): *p* = 0.017. The panels **d**-**f** show the fold changes relative to vehicle-treated samples (means±S.D.; *p < 0.05) of the most discriminant metabolites between the two groups from the PCA models (loadings ≥0.8). “c” and “p” for each extracellular metabolite indicate consumption or production, respectively

**Fig. 5:**
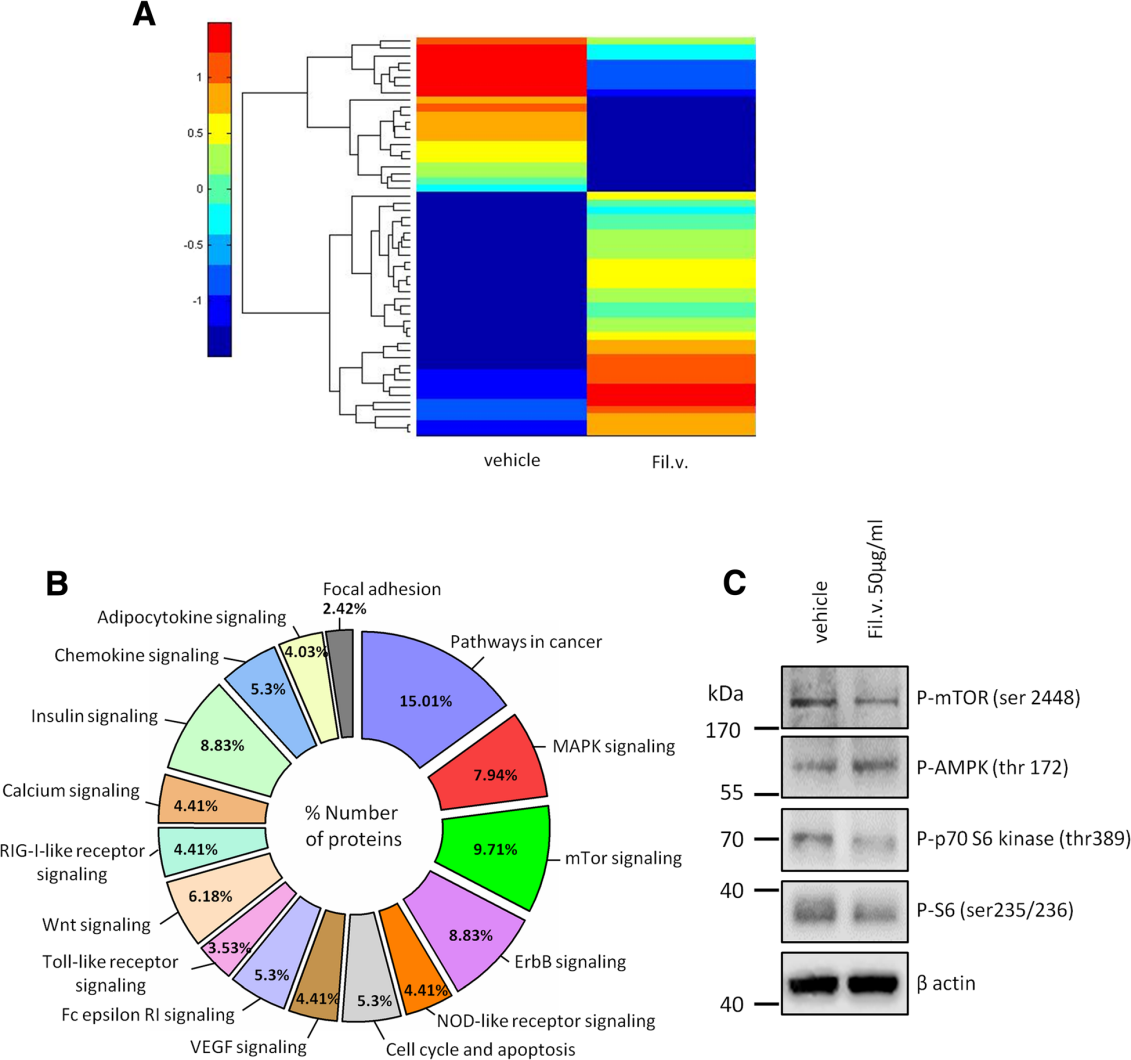
Dropwort treatment affects phosphoprotein expression profile of MSTO-211H cell lines. **a** Heatmap overall intensities of protein and phospho-protein level of MSTO-211H treated or not with Fil.v. extract (50 μg/ml). Red and blue shadings indicate high and low or undetectable protein levels respectively. **b** In silico prediction of the pathways affected by *Filipendula vugaris* extract and related to phosphoprotein perturbation (https://david.ncifcrf.gov). **c** Representative protein gel blot of whole cell lysates obtained from MSTO-211H cells treated for 24 h with 50 μg/ml of Fil.v. extract and stained with the indicated antibodies. Actin staining was used as loading control

The effectiveness of a natural agent or a standardized plant extract might reside in its complexity. Several active compounds that work in unison may simultaneously trigger several signaling pathways, thus potentially bypassing most of the mechanisms that underlie acquired resistance of cancer cells [61, 62]. Therefore, to discover which of the known signaling pathways are triggered by the Fil.v. extract, we applied two different high-throughput technologies. First, we assessed whether the Fil.v. extract changed MPM cells metabolic profile (Fig. 4). To this end, we analyzed the changes of extracellular and intracellular metabolites of MSTO-211 cells using ^1^H-NMR-based metabolomics (Fig. 4a-c). We explored the NMR data through PCA: a statistically significant separation between the treated and vehicle samples was observed on PC1 for media samples (*p* = 0.001) and hydrophilic cell extracts (*p* = 0.01) and on PC1/PC3 plane for lipophilic cell extracts (*p* = 0.017). Analysis of the loading plots on the discriminant PCs revealed that Fil.v. extract altered many features of cancer metabolism such as glucose uptake, glycolysis, Tricarboxylic Acid Cycle (TCA) e, anaplerosis and lipid metabolism (Fig. 4d-f). From exo-metabolomic analysis, we observed statistical differences in consumption and production of several metabolites of glycolysis/TCA between treated and untreated cells. Fil.v. extract caused a lower consumption of glucose, pyruvate and succinate as well as a lower production of acetate. In addition, the analysis of intracellular hydrophilic metabolites suggested a variation in the same metabolic pathways: higher concentrations of citric acid, adenosine 5′-triphosphate and myo-inositol as well as lower concentration of lactic acid and fumaric acid in treated cells with respect to control ones. It is noteworthy that Fil.v. extract induced higher consumption of amino acids (except glutamate); in particular arginine concentration in culture medium of treated cells was very low. The analysis of intracellular lipophilic metabolites showed that treated cells had lower concentration of free cholesterol, cholesteryl esters and unsaturated fatty acids as well as higher level of triacylglycerides with respect to control cells, indicating perturbations in lipid metabolism. **(**Fig. 4d-f). Subsequently, protein lysates of MSTO-211H cell treated either with vehicle or Fil.v. extract (50 μg/ml) for 24 h were loaded on a phospho-antibody array containing 1318 antibodies representative of over than 30 signaling pathways involved in cancer (Fig. 5). The Fil.v. extract-treated cells showed several differences in protein expression levels compared to the untreated ones (Fig. 5a and Additional file 2: Figure S2a). In silico prediction analysis revealed that the Fil.v. extract modified the expression of proteins involved in cancer pathways (Fig. 5b). The mammalian target of rapamycin (mTOR) signaling pathway ranked as the most significant one compared to the other pathways. mTOR pathway finely tunes cell growth, survival and metabolism in response to growth factors and nutrients stimuli [63]. It has also been reported that the PI3K/PTEN/AKT/mTOR signaling is constitutively activated in a large number of tumors including MPM [64]. To validate that the Fil.v. extract affected mTOR signaling, we checked the phospho-protein levels of mTOR and of two of its major downstream effectors, p70S6 kinase and S6 kinase. As showed in Fig. 5c, MSTO-211H cells exhibited a reduction in the phospho protein levels of mTOR, p70S6 and S6 after 24 h of treatment with 50 μg/ml of Fil.v. extract. Moreover, Fil.v. extract treatment increased the phopsho-protein levels of a well-known energy sensor protein, such as AMPK. Remarkably, its activities are known to impinge on the mTOR signalling pathway and on the Hippo pathway as well. Altogether these findings highlight a broader anti-tumoral activity of the Fil.v. extract on mesothelioma cell lines. We consider that in addition to the regulatory function of Fil.v. extract on the Hippo pathway, the extract also regulates the mTOR pathway and the metabolic profile of treated cells to make them less malignant.

### Dropwort impairs YAP and TAZ activity in MSTO-211H cells

**Fig. 6:**
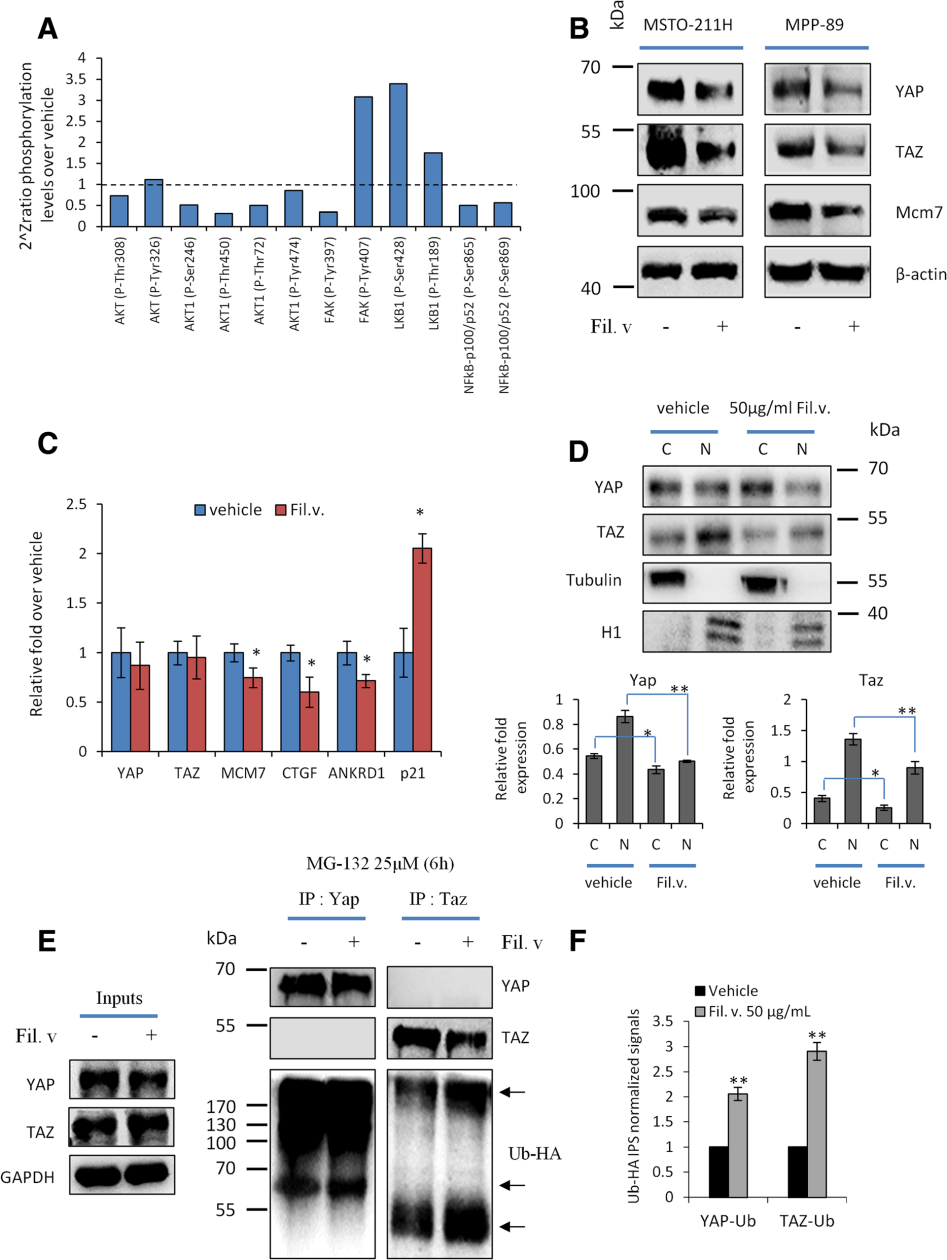
Dropwort extract modulates YAP/TAZ activities. **a** Histograms showing the 2^Zratio phosphoprotein levels of 12 selected protein known to modulate YAP and TAZ activities. This protein expression was found modulated in MSTO-211H cells treated for 24 h with Fil.v. extract compared to untreated ones (dashed line) (**b**) Representative protein gel blot of whole cell lysates obtained from MSTO-211H (left side) and MPP-89 (right side) cells treated for 72 h with 50 μg/ml of Fil.v. extract and stained with the indicated antibodies. Actin staining was used as loading control. **c** Quantitative-PCR. Histograms show the relative level of the indicated genes in MSTO-211H cells treated or not for 72 h with the Fil.v. extract. Bars indicate the average of three independent experiments. Statistics (t-test): *p* < 0.05. **d** Upper part: representative protein gel blot of nucleus/cytosol cell lysates obtained from MSTO-211H cells treated as for panel B and stained with the indicated antibodies. Tubulin and H1 staining was used as loading control for cytosol fraction and nuclei fraction respectively. Lower part: quantitative densitometry of YAP (left side) and TAZ (right side) normalized to the loading control. Bars indicate the average of three independent experiments. Statistics (t-test): *p* < 0.05. **e** MSTO211-H cells were treated or not with Fil.v. (50 μg/ml), after 24 h cells were transfected with HA-tagged Ubiquitin for further 24 h in combination with MG-132 (25 μM) for 6 h. Protein lysates were then subjected to immunoprecipitation using anti-IgG, anti-Yap or anti-Taz antibodies and subjected to Western Blotting with the indicated antibodies (right side). Arrows indicate ubiquitinated proteins (Ub-HA). Inputs recovered from anti-IgG IPs were loaded onto gels and subjected to immunoblot together with IP samples (left side). **f** Quantitative densitometry of the abundance of ubiquitinated proteins is reported in (**e**)

To dissect mechanistically the antitumoral activities of the Fil.v. extract on mesothelioma cells we assessed its impact on the key players of the Hippo pathway. Several lines of evidence have highlighted a cross-talk between the PI3K-AKT-mTOR pathway and the Hippo signaling pathway in cancer and other diseases [65–69]. Moreover, recent findings revealed the role of metabolism and of the AMPK in the modulation of YAP/TAZ activity [70]. YAP and TAZ are transcriptional co-activators that interact with the TEAD family of transcription factors and their aberrant activity leads to unrestrained cell proliferation, in several cancers types, including MPM. In MPM, the onco-suppressive Hippo regulator NF2 is often inactivated, while the oncogenic effectors YAP and TAZ are aberrantly activated [67, 71]. Accordingly, we aimed to test whether Fil.v. extract could affect the abundance and the activation status of YAP and TAZ in mesothelioma. To this end, we firstly measured the activation status of several proteins known to be involved in YAP/TAZ regulation by analysing data obtained from phospho-array assay (Fig. 5a). In agreement with the observed inhibition of the mTOR signaling pathway, we found a reduction in the phosphorylation levels of AKT (Fig. 6a) [72, 73]. Furthermore, we observed a similar reduction of phosphorylation on NF-KappaB on Ser-865 and Ser-869 and of the Focal Adhesion Kinase (FAK) on p-Tyr-397 (Fig. 6a). Conversely, the inhibitory phosphorylation on the Tyr-407 residue of FAK was increased (Fig. 6a) [74, 75]. Finally, we observed a strong increase of LKB1 phosphorylation. Several previous reports showed that AKT, NF-KappaB and FAK activate, while LKB1 inhibits, the oncogenic transcriptional co-activators YAP and TAZ, the final effectors of the Hippo signaling transduction pathway [76–79]. While Fil.v. extract treatment did not affect YAP/TAZ transcript levels (Fig. 6c), it did affect YAP and TAZ protein abundance (Fig. 6b) thereby suggesting a post-translational regulatory mechanism. Furthermore Fil.v. extract treatment down-regulated transcript and and expression levels of known YAP/TAZ/TEAD transcriptional targets such as MCM7, CTGF, ANKRD1 (Fig. 6b, c) [80–83]. Unlike HIPPO gene targets, p21waf1 expression was up-regulated thereby indicating reduction of cell proliferation (Fig. 6c).

Although the anti-tumoral activity of Fil. v. extract is likely maximized by its intrinsic complexity, to find out which components might be responsible for Fil. v. effects, we treated MSTO-211H cells with either Fil. v. total extract or single synthetic components of the extract, such as Salicylic, Chlorogenic, Gallic acid and Quercetin (Table 1 and Additional file 3: Figure S3a). Among them, we found that Gallic acid and Quercetin affected cells viability, while Salicylic and Chlonogenic acid did not impair MSTO-211H vitality (Additional file 3: Figure S3a). Consequently, we assessed whether Gallic acid and Quercetin inhibited YAP and TAZ protein levels. Unlike Gallic acid, Quercetin inhibited YAP and TAZ protein levels (Additional file 3: Figure S3b) in MSTO-211H cells. Moreover, we found that Quercetin and Fil. v. impaired YAP and TAZ protein levels in MPP-89, H-28 and H-2052 MPM cell lines (Additional file 3: Figure S3c).

Post-translational modifications of YAP and TAZ are mainly related to their cytoplasmic distribution or their degradation mediated by the proteasome pathway [84]. Thus, we firstly tested whether Fil.v. extract induced a different nucleo-cytoplasmic distribution of YAP and TAZ proteins. We observed that upon Fil.v. extract treatment the reduction of YAP and TAZ occurred mainly in the nucleus (Fig. 6d and Additional file 4: Figure S4a), but concomitantly we did not find any re-localization of YAP and TAZ from the nucleus to the cytoplasm (Fig. 6d and Additional file 4: Figure S4a). Strikingly, we observed that Fil.v. extract induced an increased ubiquitination of both YAP and TAZ proteins (Fig. 6e, f), thus suggesting that the treatment with the plant extract impaired YAP/TAZ oncogenic activities by promoting their degradation.

### Inactivation of YAP/TAZ/TEAD axis favors dropwort extract anticancer activity

**Fig. 7:**
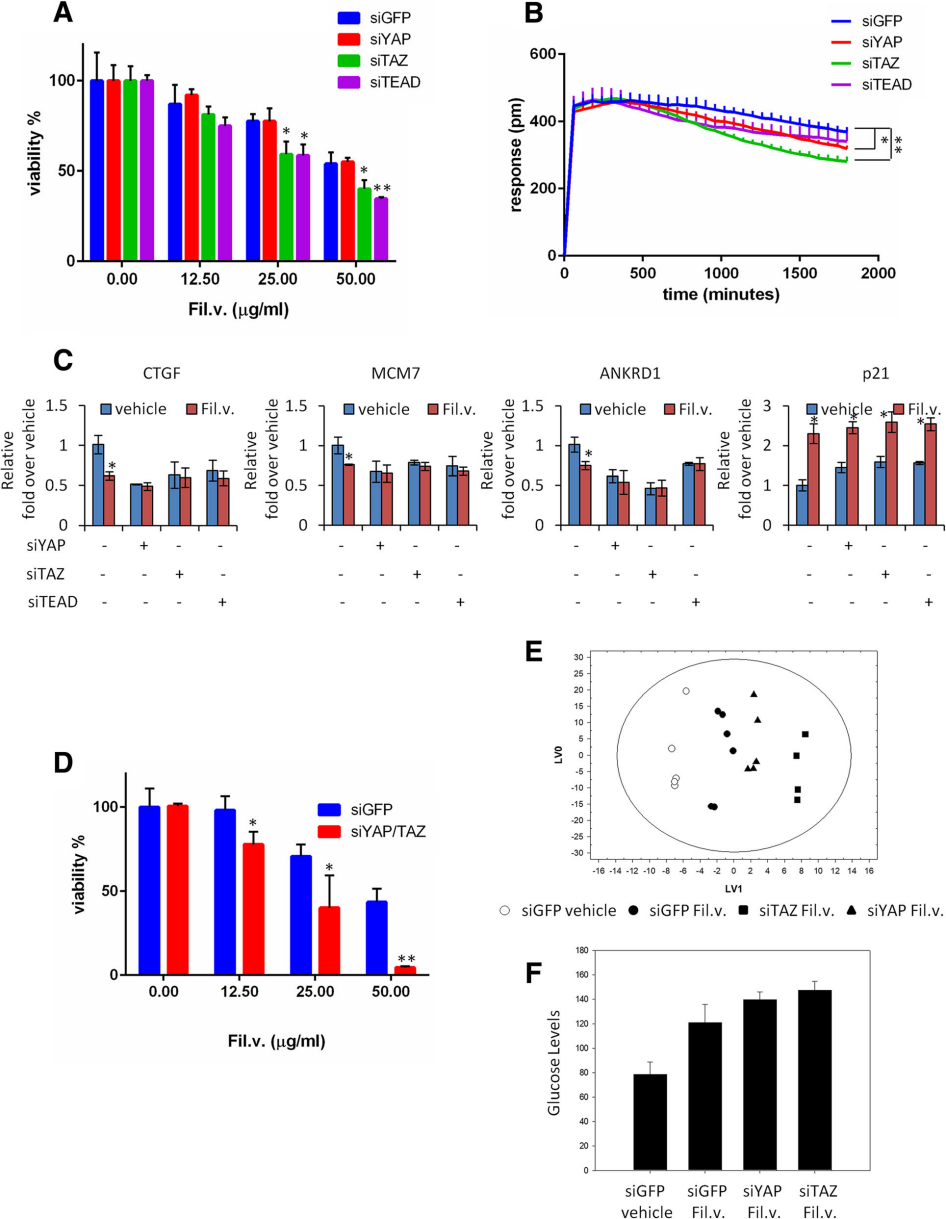
Depletion of YAP and/or TAZ affects the response of MPM cells to Dropwort extract. **a** Histograms showing the average percentage of viability of MSTO-211H cells transfected with a siRNA control or with a siRNA against YAP, or TAZ, or TEAD and treated with different doses of Fil.v. extract for 72 h as indicated. Statistics (t-test): *p* < 0.05. **b** Graphs indicating the fitness (impedance) of MSTO-211H cells transfected as from (**a**) and subsequently treated with Fil.v. extract (50 μg/ml) for 0–2000 min. Statistics (t-test): *p* < 0.05. **c** Quantitative-PCR. Histograms showing the relative level of the indicated genes in MSTO-211H cells treated as from (**a**). Bars indicate the average of three independent experiments. Statistics (t-test): *p* < 0.05. **d** Histograms showing the average percentage of viability of MSTO-211H cells transfected with a siRNA control or concurrently with siRNA against YAP and TAZ and treated with different doses of Fil.v. extract for 72 h as indicated. Statistics (t-test): *p* < 0.05. **e** Metabolic response of MSTO-211H cell lines differently depleted for siRNA control or YAP or TAZ and treated with 50 μg/ml of Fil.v. extract for 24 h. PCA models built on the ^1^H-NMR dataset of media samples. **f** Histogram show the average percentage of glucose levels obtained from analysis of the variable importance in projection (VIP) of MSTO-211H cells depleted and treated as from **e**

To further assess the contribution of the inhibition of YAP and TAZ oncogenic activities as one of the potentially leading molecular mechanisms that underlie the anticancer effects of Fil.v. extract on MPM, we tested viability of MSTO-211H cells depleted for YAP or TAZ or TEAD expression (Additional file 4: Figure S4b). Cells expressing low levels of TAZ or TEAD were more prone to Fil.v. extract-induced cell killing than control cells (Fig. 7a, b). CTGF, ANKRD1 and MCM7, but not p21, mRNAs level were not longer affected by the Fil.v. extract treatment in cells depleted of YAP, TAZ or TEAD protein expression (Fig. 7c). Conversely, we found that elevated expression of YAP or TAZ or TEAD rendered MSTO-211H cells more resistant to Fil.v. extract treatment than control cells (Additional file 4: Figure S4c-e). Moreover, ectopic expression of YAP or TAZ or TEAD rescued the Fil.v. extract-induced reduction of CTGF, ANKRD1 and MCM7 mRNA expression (Additional file 4: Figure S4f). Importantly, mRNA p21 levels were still affected by Fil.v. extract treatment independently of the YAP, TAZ or TEAD status; thus supporting the broad effect of this natural agent. We also noted that MSTO-211H cells that were depleted for both YAP and TAZ were more sensitive to Fil.v. extract treatment than control cells (Fig. 7d). In sum, these findings document that the standardized extract from *Filipendula vulagaris* plant (Fil.v. extract) exerts its anticancer effects on MPM by curtailing the oncogenic activity of the YAP/TAZ/TEAD Hippo axis. It has been reported that the activity of the YAP/TAZ transcriptional co-activators is regulated by metabolic pathways, such as mevalonate synthesis and aerobic glycolysis, and by the nutrient-sensing LKB1–AMPK and TSC–mTOR pathways [70]. The intracellular or extracellular availability of sterols and fatty acids regulate activation of SREBP transcription factors, which control the transcription of HMGCR and other enzymes of the mevalonate pathway. Thus, these metabolic intermediates are required for YAP/TAZ activity. Intracellular metabolomic analysis of lipophilic phase metabolites revealed lower levels of cholesterol and unsaturated fatty acids in Fil. v extract-treated cells with respect to control ones (Fig. 4f), suggesting a downregulation of mevalonate pathway. Moreover, it has been observed that inhibition of glucose uptake and of glycolysis induces a corresponding inhibition of YAP/TAZ activity in human cultured cells. From the analysis of extracellular metabolites, we observed that Fil. v. induced a lower consumption of glucose while the analysis of intracellular hydrophilic metabolites, confirmed a reduction of glycolytic flux in treated cells as suggested by the lactate decrease (Fig. 4d, e). Most likely, as the glucose metabolism can no longer contribute to the overall levels of ATP, AMPK could promote the activation of alternative catabolic pathways to re-establish ATP levels, thus maintaining energy homeostasis. Accordingly, the Fil. v.-treated cells showed higher degradation of amino acids as leucine, isoleucine, arginine, lysine, glutamine, methionine, tyrosine, histidine and phenylalanine with respect to control cells (Fig. 4d). Very low levels of 2-oxo-methyl-isovalerate and 2-oxo-isovalerate (data not shown) indicate a higher flux through branched-chain amino acid aminotransferase pathway compared to the control cells. As branched-chain amino acid consumption increases, the release of their catabolic intermediates decreases. This indicates that treated cells convert isoleucine and valine to succinyl-CoA preferentially. The positive correlation pattern between branched-chain amino acids and the others suggested that treated cells use amino acids for energy production, instead of macromolecule biosynthesis. Based on these metabolic data and the recent publications on the role of the HIPPO pathway on cancer metabolism [70, 85], we explored whether MSTO-211H cells depleted for YAP or TAZ protein responded differently to Fil.v. extract. Notably, the NMR data of siGFP Vehicle, siGFP Fil.v., siTAZ Fil.v. and siYAP Fil.*V. medium* cultures through OPLS-DA revealed a statistically significant separation between the treated and control samples on LV1. In particular, silencing of YAP and TAZ caused increased effects of Fil.v. on MSTO-211 cells (Fig. 7e). Moreover, analysis of the variable importance in projection (VIP) showed that the most relevant variable was glucose metabolite (VIP value = 2.24). Indeed, the inhibition of glucose uptake from Fil.v extract, was increased upon depletion of YAP/TAZ (Fig. 7f).

## Discussion

**Fig. 8:**
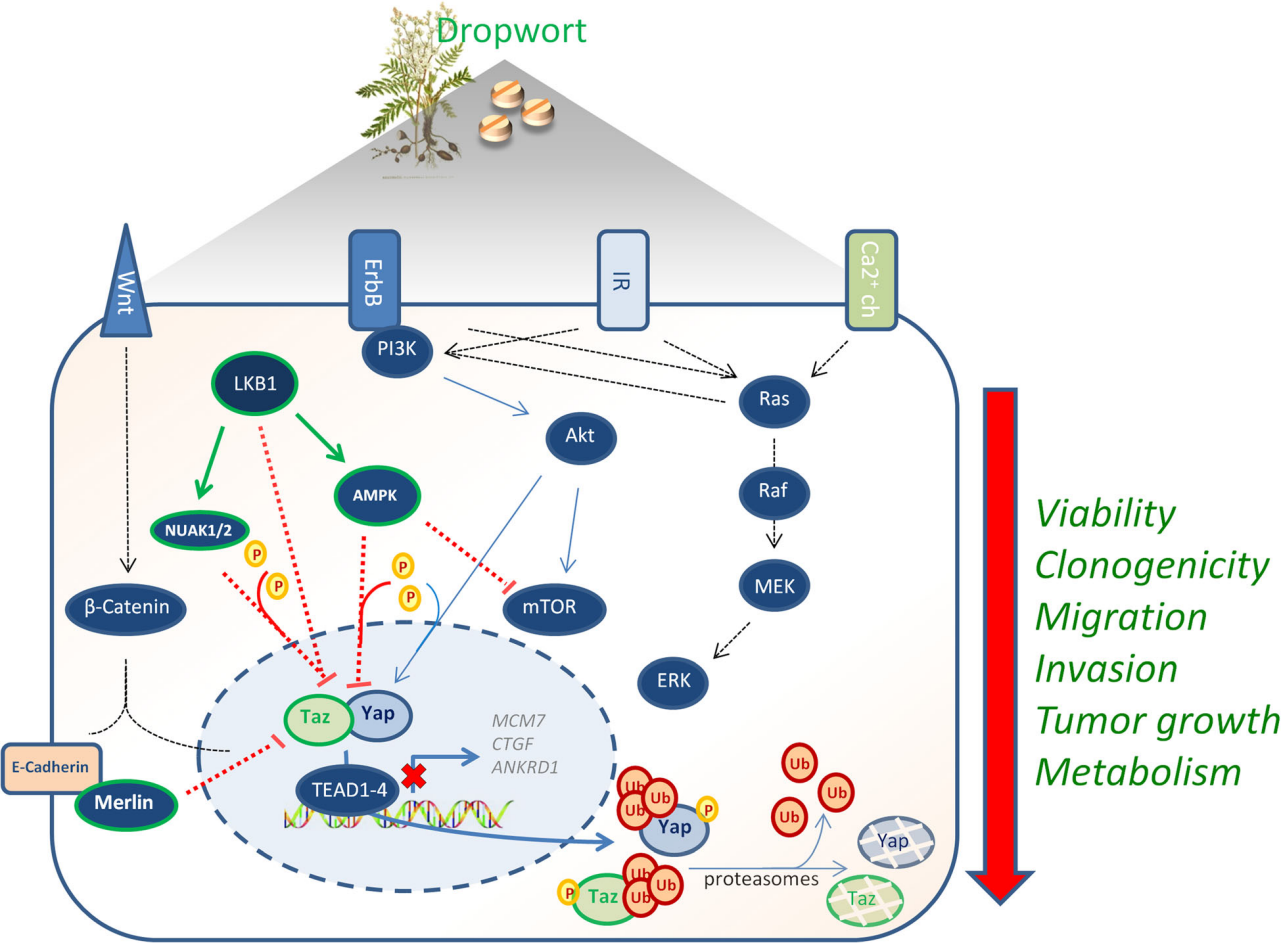
Dropwort extract exerts broad anticancer effects on MPM cells affecting cell proliferation, viability, migration, metabolism and in vivo tumor growth of MPM cell lines. This occurs by impairing simultaneously several pathways commonly deregulated in human cancers. In particular the PI3K/PTEN/AKT/mTOR signaling and the LKB1/AMPK axis indicated with blue arrows and green arrows, respectively. This perturbation leads to the Hippo pathway inhibition through the ubiquitin-mediated degradation of its oncogenic transducers, YAP and TAZ. Green and red arrows indicate activation and inhibition, respectively

We have reported here that a standardized extract from top flower of a medicinal plant, Dropwort (Fil.v. extract), was able to exert anti-tumoral activity in “in vitro” and “in vivo” models of MPM. The MPM is difficult to treat and the survival of MPM patients is very poor. Therefore, we concentrated our efforts on repurposing medicinal plants in hope of finding activities that would attenuate any aspect of MPM cancer phenotype. The broad activity and apparent efficacy of natural products,such as the components of Fil.v. extract may reside in their structural complexity and their ability to modulate multiple signaling pathways [61, 62] whose intrinsic balance impedes MPM. Mechanistically, activation of tumor suppressor pathways promoting cell death and curtailing of oncogenic signaling involved in the chemoresistance of a given tumor, would surely represent the major objective to fulfill for treating successfully MPM. We documented here that Fil.v. extract-treatment of MPM cells triggered simultaneously diverse signaling pathways commonly deregulated in human cancers (Fig. 8). Fil.v. extract impaired aberrant activation of mTOR and of its downstream effectors. This paired well with a global modulation of main components of the metabolic profiling of MPM cells. Emerging lines of evidence link metabolic alterations and mTOR aberrant activation with sustained pro-tumorigenic activities of the HIPPO tumor suppressor pathway [65–68, 70]. MPM is characterized by a relatively low gene mutational rate in which several key members of the HIPPO tumor suppressor pathway represent an exception. The example of the mutated gene in MPM is NF2**,** an upstream tumor suppressor regulator that couples frequently with the loss of LATS2 expression, a pivotal member of the core of the HIPPO pathway in MPM [33, 35–37, 40–42]. This double inactivation of two tumor suppressors leads to the aberrant activation of the two HIPPO oncogenic transducers, YAP and TAZ, which enhance the transcriptional activity of TEAD family of potent transcription factors [41, 42]. Excessive nuclear accumulation of YAP/TAZ is critical for its oncogenic co-transcriptional activity; thereby compounds that re-localize YAP/TAZ to the cytoplasm strongly impair their pro-tumorigenic activities in various cancers [86, 87]. Notably, Fil.v. extract promotes ubiquitination of both YAP and TAZ and thus fully curtails their critical oncogenic potential. Indeed, depletion of YAP or TAZ or both favors Fil.v. extract-induced cell death of MPM cell lines. Further evidence on the effect of Fil.v. extract treatment on the HIPPO pathway is provided by the downregulation of YAP/TAZ-TEAD transcriptional targets such as CTGF, MCM7, ANKRD1 genes. In sum, these findings demonstrate that Fil.v. restrains the oncogenic activity of the HIPPO pathway through the hampering of the transcriptional axis YAP/TAZ/TEAD in MPM (Fig. 8).

We previously reported that MPM cells with elevated ALDH activity endow with tumor initiating features, thus contributing strongly to in vitro and in vivo chemoresistance of MPM [3]. Fil.v. treatment affects ALDH-bright MPM cells, thus it might hold the potential to tackle actively MPM subcellular populations highly chemoresistant. At least, at the preclinical stage, Fil.v. synergizes with both cisplatin and Pemetrexed and reduces the volume of MPM xenografted tumors as efficienttly as Pemetrexed. As a whole, these findings support the possibility that Fil.v. might complement and reinforce either cisplatin- or Pemetrexed-induced anticancer effects on MPM cells. Furthermore, Fil.v. exhibits the ability to tackle MPM tumor initiating cells, thereby having at the preclinical stage a profound cell killing effect on highly resistant cell subpopulations of MPM.

## Conclusions

MPM is still a poorly treated tumor. Either novel therapeutic approaches or the repurposing of existing drugs might contribute to tackle this unmet clinical need. Despite the apparent biochemical complexity of plant extracts that are derived from *Filipendula vulgaris*, for the specific formulation of Fil.v. extract used here, an anticancer activity could be documented in several cell culture and animal models of MPM. It would be important now to identify the complete mechanism via which the extract achieves anti-tumoral properties in order to candidate it to become a potent drug for the MPM treatment. Our previous published natural compound derived from leaf extracts of artichoke is actually in clinical trial (NCT 02076672) for the treatment of subjects with asbestosis [25] . This leads the way to the potential clinical application of *Filipendula vulgaris* either alone or in combination with leaf extracts of artichoke.

## Additional files

Additional file 1: **Figure S1.** (**a**-**b**) Percentage of the four different sub cells population of MSTO-211H (**a**) and MPP-89 (**b**) cells treated with the indicated doses of the Fil.v. extract for 72 h. Error bars represent mean +/− SD. Statistics (t-test): *p* < 0.05. (**c**) IC-50 value calculated by compusyn software obtained by treating the indicated cancer cell lines with different doses of Fil.v. (0–200 μg/ml) for 72 h. (**d**-**e**) Histograms show the percentage of Annexin V+/PI- over vehicle. MSTO-211H (**d**) or MPP-89 (**e**) cells were treated at the indicated doses of Fil.v. extract for 24 h. Error bars represent mean +/− SD. Statistics (t-test): *p* < 0.05. (**f**) Representative protein gel blot of whole cell lysates obtained from MSTO-211H cells treated for 24 h with 100 μg/ml of Fil.v. extract and probed with the indicated antibodies. Actin staining was used as loading control. (TIF 1250 kb)

Additional file 2: **Figure S2.** (**a**) List of all proteins and phospho-proteins that result deregulated after 24 h of Fil.v. extract treatment. Red and blue colors indicate up and down regulation respectively. (TIF 748 kb)

Additional file 3: **Figure S3.** (**a**) Viability of MSTO-211H cell lines treated for 72 h with either different doses of Fil.v. extract (0-200 μg/ml) or synthetic Salicylic acid (0–1.08 μg/ml), Chlorogenic acid (0–2.24 μg/ml), Gallic acid (0–19.98 μg/ml) or Quercetin (0–26.86 μg/ml). (**b**-**c**) Representative protein gel blot of whole cell lysates obtained from (**b**) MSTO-211H or (**c**) MPP89, H-28 and H-2052 cells treated as indicated and stained with the indicated antibodies. (TIF 1548 kb)

Additional file 4: **Figure S4.** (**a**) Immunofluorescence of MSTO-211H cells treated or not with Fil.v. extract, 50 μg/ml, and stained with anti-YAP and TAZ antibodies. Nuclei were stained with DAPI. Scale bar, 20 μm. (**b**-**c**) Representative protein gel blot of whole cell lysates obtained from MSTO-211H cells treated (**b**) as for Fig. 6a or (**c**) as for Supplementary Fig. S4d and stained with the indicated antibodies. Actin staining was used as loading control. (**d**) Histograms show the average percentage of viability of MSTO-211H cells expressing either a control vector or a YAP or a TAZ or a TEAD expressing vector and treated with different doses of Fil.v. extract for 72 h as indicated. Statistics (t-test): *p* < 0.05. (**e**) Graphs indicating the fitness (impedance) of MSTO-211H cells transfected as from (**d**) and subsequently treated with Fil.v. extract (50 μg/ml) for 0–2000 min to evaluate early changes in cell fitness, as assessed by a label free assay. Statistics (t-test): *p* < 0.05. (**f**) Quantitative-PCR. Histograms showing the relative level of the indicated genes in MSTO-211H cells treated as from (**a**). Bars indicate the average of three independent experiments. Statistics (t-test): *p* < 0.05. (TIF 2604 kb)

## Data Availability

All data generated or analyzed during this study are included in this published article. Raw and processed data are stored in the laboratories of SS and GB and are available upon request.

## Abbreviations

ALDH: Aldehyde Dehydrogenase
BAP1: associated protein gene 1
CDDP: Cisplatin
CDNKN2A: cyclin dependent kinase inhibitor 2A gene
EMT: Epithelial-To-Mesenchymal Transition
FAK: Focal Adhesion Kinase
Fil.v.: *Filipendula vulgaris*
HMC: human untransformed mesothelial cell
MPM: malignant pleural mesothelioma
mTOR: mammalian target of rapamycin
NF2: Neurofibromin 2
PMTX: Pemetrexed
TCA: Tricarboxylic Acid Cycle
VIP: variable importance in projection
