# Supplementary figures and images for: Dropwort-induced metabolic reprogramming restrains YAP/TAZ/TEAD oncogenic axis in mesothelioma
Source: J Exp Clin Cancer Res. 2019 Aug 9;38:349. doi: 10.1186/s13046-019-1352-3 (PMC6689183; doi:10.1186/s13046-019-1352-3)

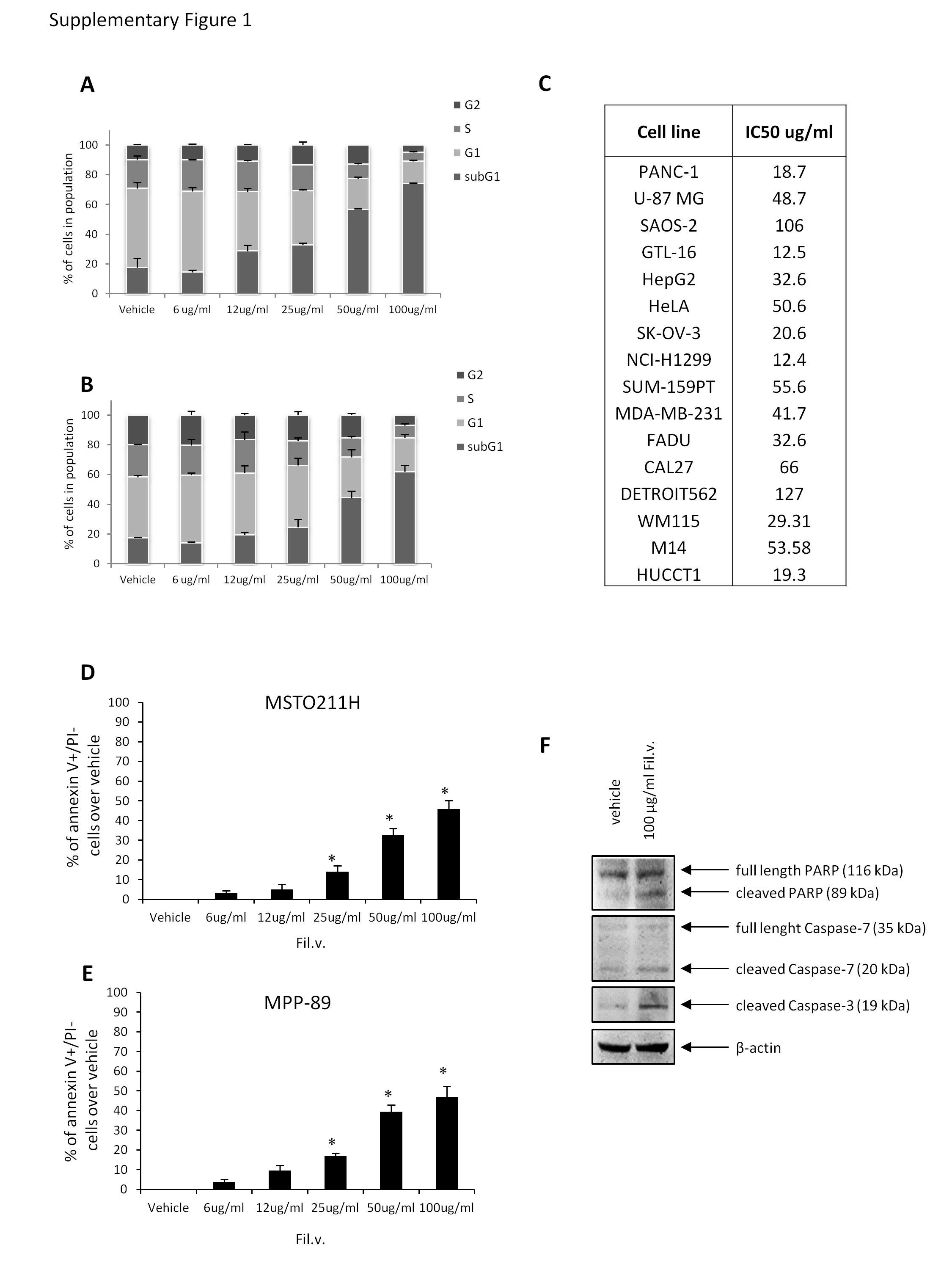

Supplement: Supplementary file 1 — Figure S1. (a-b) Percentage of the four different sub cells population of MSTO-211H (a) and MPP-89 (b) cells treated with the indicated doses of the Fil.v. extract for 72 h. Error bars represent mean +/− SD. Statistics (t-test): p < 0.05. (c) IC-50 value calculated by compusyn software obtained by treating the indicated cancer cell lines with different doses of Fil.v. (0–200 μg/ml) for 72 h. (d-e) Histograms show the percentage of Annexin V+/PI- over vehicle. MSTO-211H (d) or MPP-89 (e) cells were treated at the indicated doses of Fil.v. extract for 24 h. Error bars represent mean +/− SD. Statistics (t-test): p < 0.05. (f) Representative protein gel blot of whole cell lysates obtained from MSTO-211H cells treated for 24 h with 100 μg/ml of Fil.v. extract and probed with the indicated antibodies. Actin staining was used as loading control. (TIF 1250 kb) [file 13046_2019_1352_MOESM1_ESM.tif]

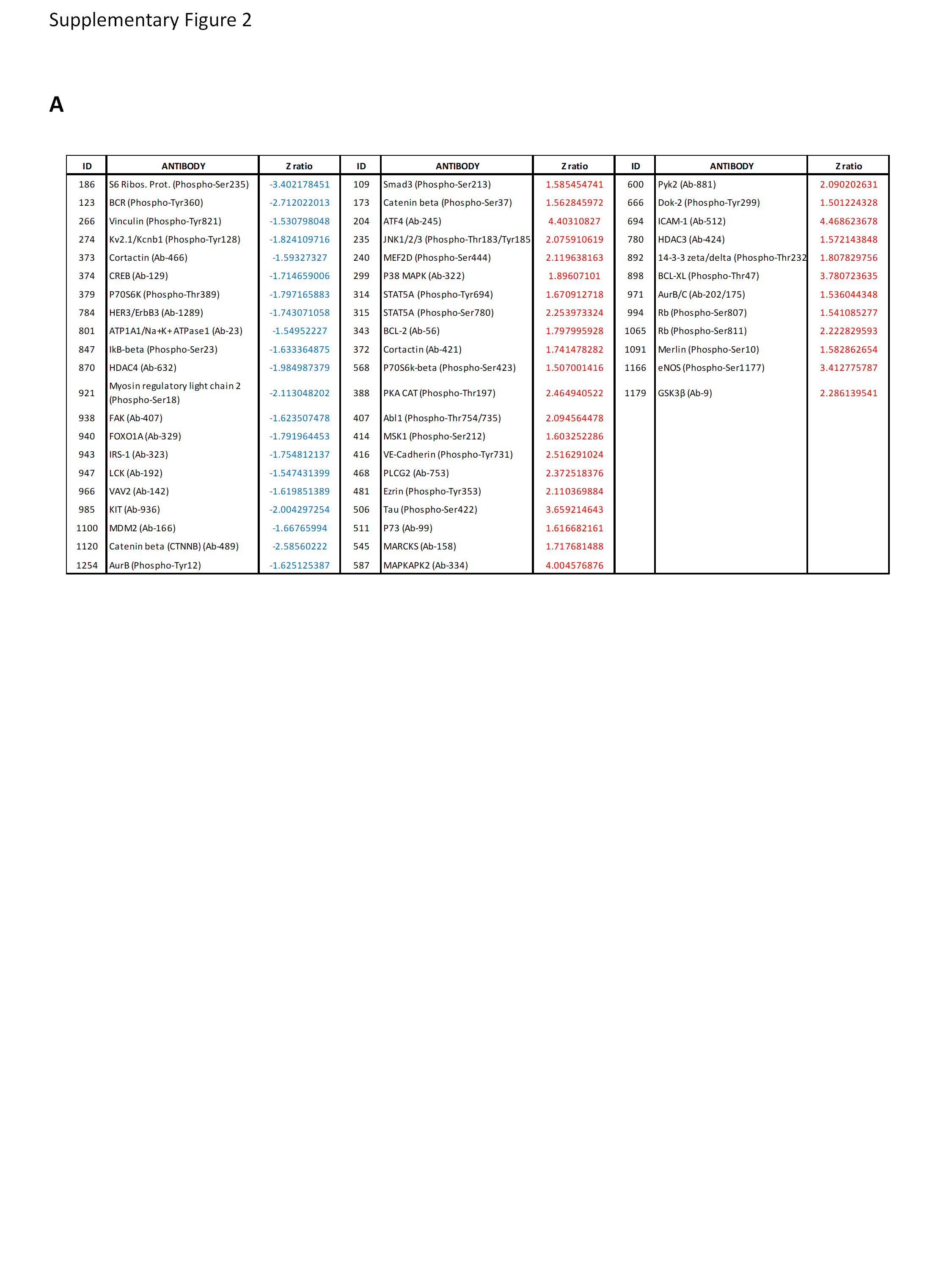

Supplement: Supplementary file 2 — Figure S2. (a) List of all proteins and phospho-proteins that result deregulated after 24 h of Fil.v. extract treatment. Red and blue colors indicate up and down regulation respectively. (TIF 748 kb) [file 13046_2019_1352_MOESM2_ESM.tif]

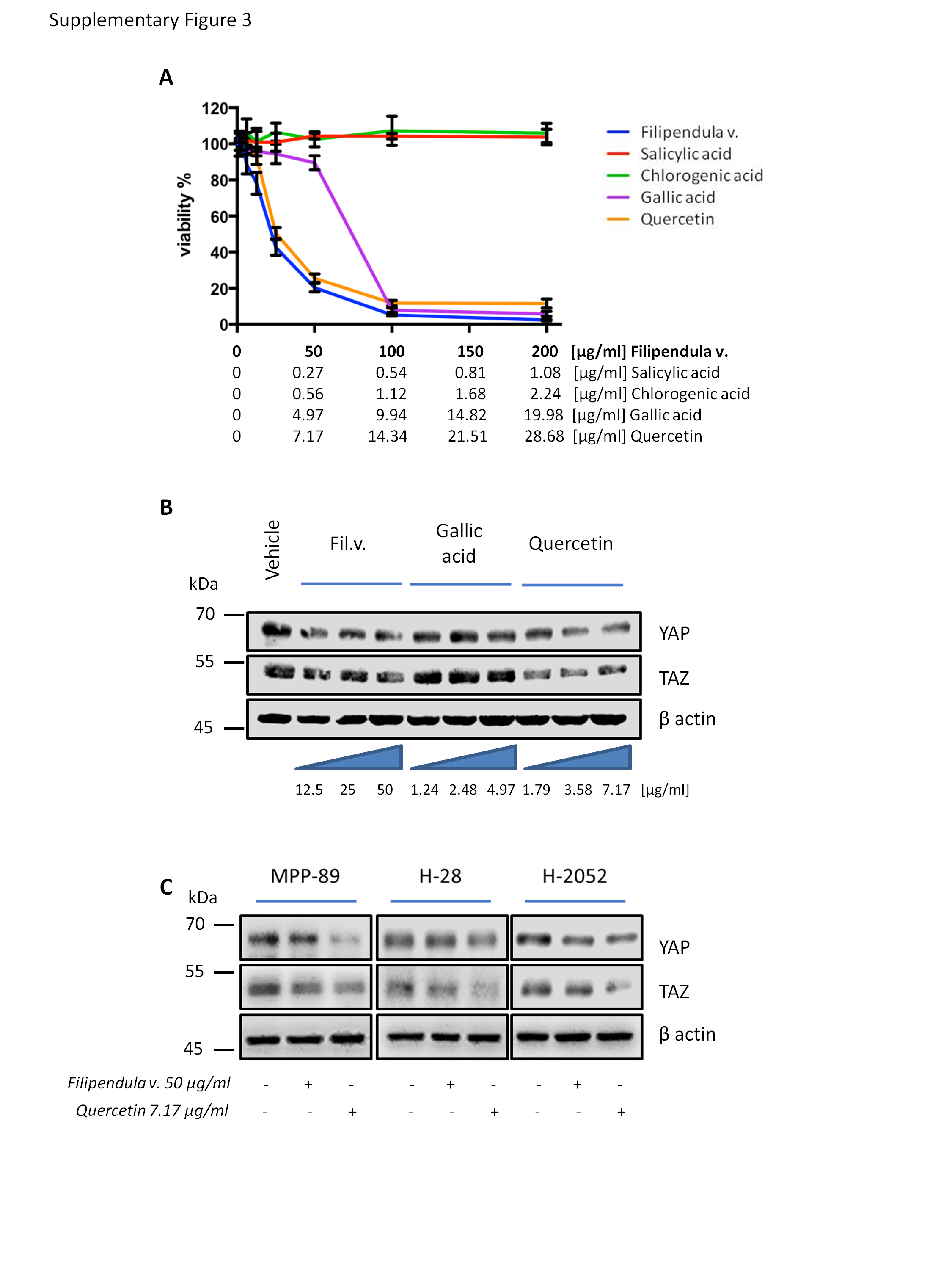

Supplement: Supplementary file 3 — Figure S3. (a) Viability of MSTO-211H cell lines treated for 72 h with either different doses of Fil.v. extract (0-200 μg/ml) or synthetic Salicylic acid (0–1.08 μg/ml), Chlorogenic acid (0–2.24 μg/ml), Gallic acid (0–19.98 μg/ml) or Quercetin (0–26.86 μg/ml). (b-c) Representative protein gel blot of whole cell lysates obtained from (b) MSTO-211H or (c) MPP89, H-28 and H-2052 cells treated as indicated and stained with the indicated antibodies. (TIF 1548 kb) [file 13046_2019_1352_MOESM3_ESM.tif]

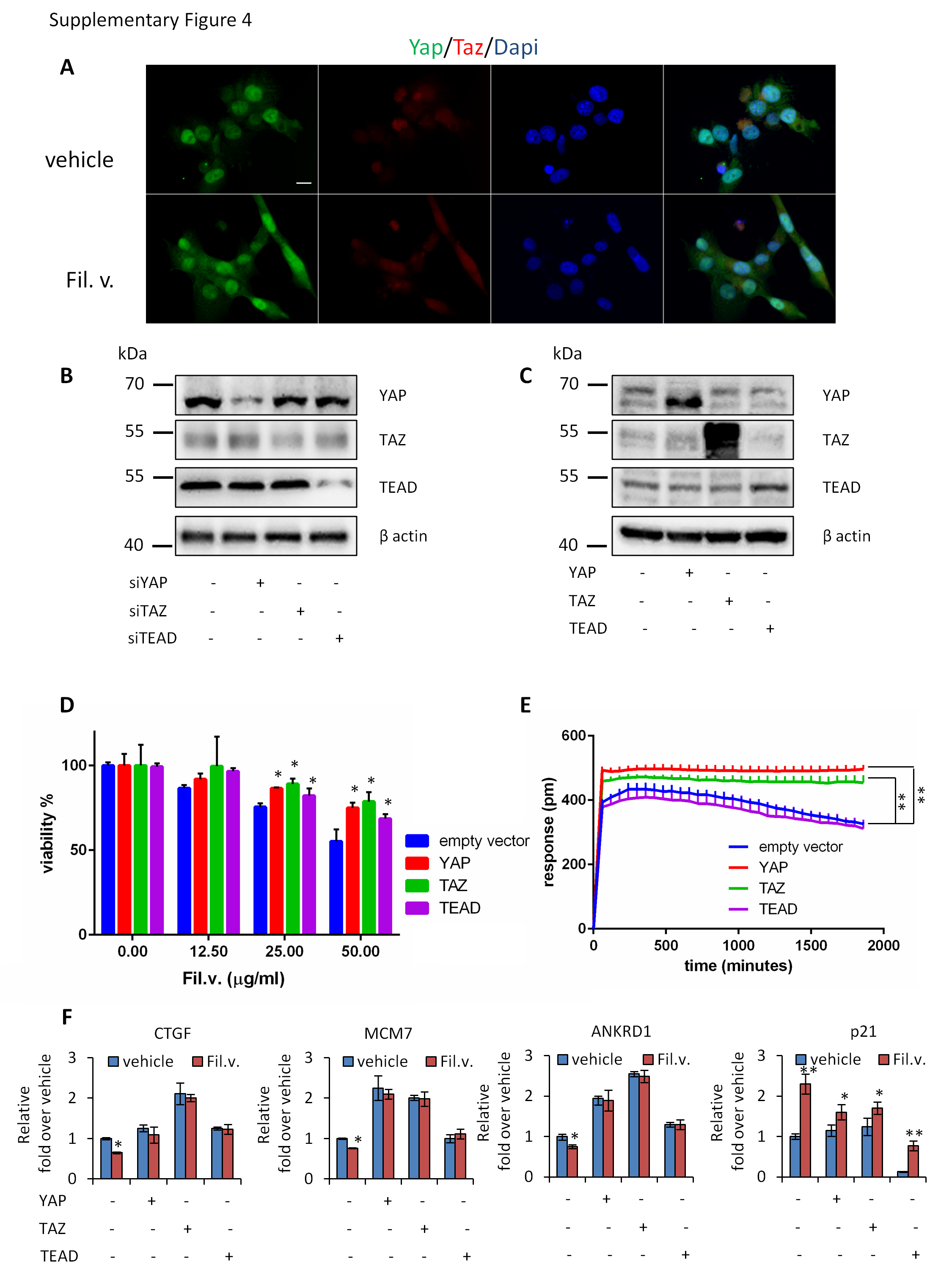

Supplement: Supplementary file 4 — Figure S4. (a) Immunofluorescence of MSTO-211H cells treated or not with Fil.v. extract, 50 μg/ml, and stained with anti-YAP and TAZ antibodies. Nuclei were stained with DAPI. Scale bar, 20 μm. (b-c) Representative protein gel blot of whole cell lysates obtained from MSTO-211H cells treated (b) as for Fig. 6a or (c) as for Supplementary Fig. S4d and stained with the indicated antibodies. Actin staining was used as loading control. (d) Histograms show the average percentage of viability of MSTO-211H cells expressing either a control vector or a YAP or a TAZ or a TEAD expressing vector and treated with different doses of Fil.v. extract for 72 h as indicated. Statistics (t-test): p < 0.05. (e) Graphs indicating the fitness (impedance) of MSTO-211H cells transfected as from (d) and subsequently treated with Fil.v. extract (50 μg/ml) for 0–2000 min to evaluate early changes in cell fitness, as assessed by a label free assay. Statistics (t-test): p < 0.05. (f) Quantitative-PCR. Histograms showing the relative level of the indicated genes in MSTO-211H cells treated as from (a). Bars indicate the average of three independent experiments. Statistics (t-test): p < 0.05. (TIF 2604 kb) [file 13046_2019_1352_MOESM4_ESM.tif]
